# Proteomic Analysis of Combined Gemcitabine and Birinapant in Pancreatic Cancer Cells

**DOI:** 10.3389/fphar.2018.00084

**Published:** 2018-02-19

**Authors:** Xu Zhu, Xiaomeng Shen, Jun Qu, Robert M. Straubinger, William J. Jusko

**Affiliations:** ^1^Department of Pharmaceutical Sciences, University at Buffalo, The State University of New York, Buffalo, NY, United States; ^2^Department of Biochemistry, University at Buffalo, The State University of New York, Buffalo, NY, United States

**Keywords:** proteomics, pancreatic cancer cells, gemcitabine, birinapant, signaling pathways, drug targets, drug resistance

## Abstract

Pancreatic cancer is characterized by mutated signaling pathways and a high incidence of drug resistance. Comprehensive, large-scale proteomic analysis can provide a system-wide view of signaling networks, assist in understanding drug mechanisms of action and interactions, and serve as a useful tool for pancreatic cancer research. In this study, liquid chromatography-mass spectrometry-based proteomic analysis was applied to characterize the combination of gemcitabine and birinapant in pancreatic cancer cells, which was shown previously to be synergistic. A total of 4069 drug-responsive proteins were identified and quantified in a time-series proteome analysis. This rich dataset provides broad views and accurate quantification of signaling pathways. Pathways relating to DNA damage response regulations, DNA repair, anti-apoptosis, pro-migration/invasion were implicated as underlying mechanisms for gemcitabine resistance and for the beneficial effects of the drug combination. Promising drug targets were identified for future investigation. This study also provides a database for systems mathematical modeling to relate drug effects and interactions in various signaling pathways in pancreatic cancer cells.

## Introduction

Pancreatic cancer is one of the most lethal malignancies, with 5-year survival of only 8% (Siegel et al., 2016). It is projected to become the second leading cause of cancer death by 2030 (Rahib et al., 2014). The extremely poor prognosis results from delayed diagnosis, early metastasis, and resistance to almost all classes of cytotoxic drugs (Cecconi et al., 2011). Pancreatic cancer is characterized by mutations in multiple signaling pathways. Comprehensive evaluation of the pancreatic cancer genome detected >1000 mutations categorized into 14 core signaling pathways, including DNA damage control and apoptosis (Jones et al., 2008; Yachida and Iacobuzio-Donahue, 2013). These pathways usually are functionally redundant, and therefore pharmacological inhibition of individual nodes in one pathway can lead to activation of alternative pathways and induce drug resistance.

The nucleoside analog gemcitabine (2′-deoxy-2′-difluorodeoxycytidine), and gemcitabine-based drug combinations, are standard treatment for pancreatic cancer. However, the survival benefit of gemcitabine alone is only 6 months (Burris et al., 1997), and the combination of gemcitabine and Abraxane^®^ (nab-paclitaxel), approved in 2013, increased average survival to just 8.5 months (Von Hoff et al., 2013). The lack of greater benefit often is related to the existence or emergence of drug resistance, which can arise through several mechanisms that have not been characterized systematically (Kim and Gallick, 2008; Hung et al., 2012). Birinapant (TL32711; Tetralogic, Malvern, PA, United States) is a bivalent investigational antagonist of inhibitor of apoptosis proteins (IAP). It mimics the action of the second mitochondria-derived activator of caspase, and has showed clinical activity in hematological malignancies and solid tumors as a single agent and in combination with other chemotherapeutics. Our previous studies with pancreatic cancer cell cultures determined that the combination of gemcitabine and birinapant is synergistic, and proposed that mechanisms responsible for these positive drug interactions relate to cell cycle progression and apoptosis signaling (Zhu et al., 2015).

Comprehensive, global protein analysis could provide the information at the effector protein level that is required to understand how cells function (Altelaar et al., 2012). Proteomics can provide system-wide views of signaling networks and assist in the understanding of drug mechanisms of action and interactions (Aebersold and Mann, 2003). Proteomics also provide the knowledge needed for identification of biomarkers and for pharmacological targeting specific protein pathways (Shruthi et al., 2016).

In this study, large-scale liquid chromatography-mass spectrometry (LC-MS)-based proteomics analysis of pancreatic cancer cells was performed to elucidate dynamic temporal changes in the proteome that encompass broad signaling pathways in a biological system perturbed by treatment with gemcitabine and birinapant as single and combined agents. A total of 4069 drug-responsive proteins were identified and quantified simultaneously with confidence over a time course of 72 h. Western blot analysis and extensive literature searching also provided supportive and validating information. The dataset generated provides insights into the mechanisms responsible for the beneficial effects of these drugs as a combination, and constitutes a valuable resource for pharmacodynamic modeling of networks. Mechanisms related to gemcitabine resistance are also explored and promising signaling pathways are proposed as drug targets.

## Experimental Procedures

### Cell Culture

The human pancreatic cancer cell line PANC-1 was obtained from the American Type Culture Collections (Rockville, MD, United States). Cells were cultured in Dulbecco’s modified Eagle’s medium (Cellgro, Manassas, VA, United States) containing 10% (v/v) fetal bovine serum (Atlanta Biological, Lawrenceville, VA, United States) in a humidified atmosphere with 5% CO_2_ at 37°C. Cells were passaged at 80–90% confluence using 0.05% trypsin with 0.53 mM EDTA (Gibco BRL, Gaithersburg, MD, United States).

### Total Protein Extraction and Quantification

Cells were seeded in T75 flasks at a density of 1 × 10^6^ cells per flask in a volume of 10 ml. After overnight incubation to allow adherence, cells were exposed to 20 nM gemcitabine, 100 nM birinapant, or combination of 20 nM gemcitabine with 100 nM birinapant. The vehicle control was DMSO at a final concentration of 0.05% (v/v), which exceeded the highest concentration present in any drug-treated wells (0.002% v/v). After exposure of triplicate samples for 0, 6, 24, 48, and 72 h, attached cells were detached using trypsin/EDTA and combined with detached cells harvested from the supernatant by centrifugation. The harvested cells were washed by centrifugation three times with 5 ml ice-cold PBS. The cell pellet was resuspended in the smallest possible volumes of ice-cold lysis buffer (containing 50 mM Tris-FA, 150 mM NaCl, 0.5% sodium deoxycholate, 2% NP-40, and 2% sodium dodecyl sulfate, pH 8.0) with Halt^TM^ Protease and Phosphatase Inhibitor Cocktail (Thermo Fisher Scientific, Rockford, IL, United States) with vigorous vortex mixing. The samples were incubated on ice for 30 min and vortexed every 10 min. Sonication was performed until the solution became pellucid in order to achieve adequate lysis and DNA shearing. The cell lysates were centrifuged at 14,000 g for 15 min at 4°C, and the supernatant was transferred to Eppendorf tubes and stored at -80°C until analysis. A Pierce^TM^ BCA Protein Assay Kit (Thermo Fisher Scientific) was used for quantification of total protein.

### Western Blot Analysis

Samples (30 μg/20 μl) were electrophoresed on NuPAGE^®^ 4–12% Bis-Tris mini gels (Invitrogen, Carlsbad, CA, United States) and transferred to PVDF membranes with the iBlot 2 transfer system (Invitrogen). The membranes were blocked in Tris-buffered saline/Tween 20 (TBST, Cell Signaling, Danvers, MA, United States) supplemented with 5% BSA or milk for 1 h. Membranes were probed overnight at 4°C with the following primary antibodies from Cell Signaling: p21 (#2947, 1:1000), cyclin D1 (#2978, 1:500), cyclin B1 (#12231, 1:500), phospho-Rb protein (#8516, 1:1000), Rb protein (#9309, 1:500), Bcl-2 (#2870, 1:1000), p65 (#8242, 1:1000), phospho-p38 (#4511, 1:500), p38 (#8690, 1:1000), PARP (#9532, 1:1000), caspase 3 (#9665). The GAPDH (#2118) and β-actin (#3700, 1:2500) were used as loading controls. After three washes with TBST, the membranes were incubated with horseradish peroxidase-conjugated anti-rabbit (#7074) or anti-mouse (#7076) IgG (Cell Signaling). Bands were developed by incubation with SuperSignal West Pico Chemiluminescent Substrate (Thermo Fisher Scientific), and detected with a ChemiDoc^TM^ MP System (Bio-Rad, Hercules, CA, United States). The bands were quantified using Image Lab 5.1 (Bio-Rad). NF-κB activity was determined by the amount of nuclear p65 relative to the cytoplasmic p65. NE-PER^TM^ nuclear and cytoplasmic extraction reagents (Thermo Fisher Scientific) was used for separation of nuclear and cytoplasmic proteins, and nuclear and cytoplasmic p65 were quantified by gel electrophoresis and western blot as described above. Anti-TATA binding protein TBP antibody (ab63766, 1:1000) from Abcam (Cambridge, MA, United States) was used to provide a loading control for nuclear proteins.

### Protein Digestion

The developed protein digestion procedure for LC-MS analysis has been described in details previously (Nouri-Nigjeh et al., 2014; An et al., 2015). One hundred micrograms of protein from each sample were transferred into individual Eppendorf tubes and reduced by addition of 5 mM dithiothreitol for 30 min. The protein mixture was further alkylated by addition of 20 mM iodoacetamide for 30 min. The reduction and alkylation of proteins were both conducted at 37°C with vigorous mixing in an Eppendorf Thermomixer (Eppendorf, Hauppauge, NY, United States) at 200 rpm. The proteins were precipitated by stepwise addition of 6 volumes of chilled acetone with continuous vortex mixing, and were incubated at -20°C overnight. After centrifugation at 20,000 *g* at 4°C for 30 min, the supernatants were discarded and the pellet containing precipitated proteins was washed with 500 μl of a chilled acetone/water mixture (85/15, v/v%) and air-dried. For on-pellet digestion, a two-step enzyme addition strategy was employed that included: (1) digestion-aided pellet dissolution, in which trypsin, at an enzyme/substrate ratio of 1:20 (w/w), was dissolved in 100 μl of Tris buffer (50 mM, pH 8.5) and added to the precipitated protein pellets, and the mixture was incubated at 37°C for 6 h with constant mixing in an Eppendorf Thermomixer; (2) complete cleavage: dissolved trypsin at an enzyme/substrate ratio of 1:20 (w/w) was added to the re-dissolved and partially cleaved proteins, and the mixture was incubated at 37°C overnight (12 h). Digestion was terminated by addition of 1% formic acid.

### Nano LC-MS/MS Analysis with a High-Field Orbitrap

The nano-RPLC (reverse-phase liquid chromatography) system consisted of a Spark Endurance autosampler (Emmen, Netherlands) and an ultra-high pressure Eksigent (Dublin, CA, United States) Nano-2D Ultra capillary/nano-LC system. Mobile phases A and B were 0.1% formic acid in 2% acetonitrile and 0.1% formic acid in 88% acetonitrile, respectively. Four micrograms of sample were loaded onto a reversed-phase trap (300 μm ID × 1 cm), with 1% mobile phase B at a flow rate of 10 μl/min, and the trap was washed for 3 min. A series of nanoflow gradients (flow rate 250 nl/min) was used to back-flush the trapped samples onto the nano-LC column (75-μm ID × 100 cm) for separation. The nano-LC column was heated at 52°C to improve both chromatographic resolution and reproducibility. A 2.5-h gradient was used to achieve sufficient peptide separation. The optimized gradient profile was as follows: 4% B over 15 min; 13–28% B over 110 min; 28–44% B over 5 min; 44–60% B over 5 min; 60–97% B in 1 min, and finally isocratic at 97% B for 17 min. An Orbitrap Fusion Mass Spectrometer (Thermo Fisher Scientific, San Jose, CA, United States) was used for MS analysis. For general analysis, the instrument was operated in the data dependent mode: MS1 spectra were collected at a resolution of 120,000, with an automated gain control (AGC) target of 500,000, and a maximum injection time of 50 ms. The *m/z* range for MS1 full scan is 400–1500. Previously interrogated precursors were excluded using a dynamic window (60 s ± 10 ppm). Precursors were filtered by quadrupole using an isolation window of 1 Th. MS2 spectra were collected at a resolution of 15,000 in the Orbitrap, with an AGC target of 50,000, and a maximum injection time of 50 ms. Precursors were fragmented by high-energy collision dissociation at a normalized collision energy of 35%.

### Protein Identification and Quantification

The individual raw files (.raw) generated by LC-MS analysis were matched to the human database containing 23,306 entries, using the MS-GF+ searching engines (released on May 17, 2013) (Kim and Pevzner, 2014). The search parameters set were as follows: (1) precursor ion mass tolerance: 20 ppm; (2) instrument: Q-Exactive; (3) one match per spectrum is allowed; (4) fixed modification: carbamidomethylation of cysteine; (5) dynamic modification: oxidation of methionine and acetylation of N-terminal. Protein/peptide filtering and control of the false discovery rate (FDR) was accomplished in Scaffold (v4.3.2, Proteome Software Inc.) (Searle, 2010) using a target-decoy search strategy with a concatenated database containing both forward and reverse sequences (Elias et al., 2005). Both protein and peptide FDR were controlled at <1%, and a minimum of two unique peptides was required.

Quantitative data analysis in IonStar was achieved by using SIEVE and IonStar-stat. Chromatographic alignment and ion intensity-based MS1 feature detection/extraction was performed using SIEVE (v2.2, Thermo Fisher Scientific). The principal procedures in SIEVE included the following: (1) chromatographic alignment among LC-MS/MS runs using the ChromAlign algorithm (Sadygov et al., 2006). Quality control of the alignment of LC-MS/MS runs was achieved by monitoring and benchmarking the alignment scores (>0.8) and base-peak intensity; (2) feature generation: features were generated for all precursors having existing MS/MS scans and extracted ion currents in the aligned collective dataset by using user defined *m/z* width- and retention time width windows centered on the existed precursor ion; 10 ppm and 1 min were used for analysis of our datasets. The resulting feature intensities then were correlated to scan numbers with identified peptide spectrum matches (PSM), generated by Scaffold, using an in-house R script *Load.R*^1^. Then the quantitative data at the PSM level were subjected to duplicate frame removal, appropriate normalization, multivariate outlier detection/rejection, and aggregation to the protein level using an in-house R package *IonStarStat*^1^.

In order to calculate protein ratios, the average protein intensity at time 0 h was used as the control in all comparisons. The *p*-value was calculated from a one-way ANOVA test using the *anov* function of R Bioconductor across all time points in each treatment group. Proteins with >1.4-fold change and *p*-value <0.05 were considered to be differentially expressed proteins.

### Bioinformatic Analysis

Analyses of gene ontology and Kyoto Encyclopedia of Genes and Genomes (KEGG) pathways were performed using DAVID 6.7 (Database for Annotation, Visualization, and Integrated Discovery) bioinformatics tools (Huang et al., 2009; Kanehisa et al., 2012). For DAVID analysis, all human proteins were used as background proteins. Function category and upstream regulator analysis (URA) was carried out using Ingenuity Pathway Analysis (IPA) for a Core Analysis. The causal analysis approach implemented in IPA was described previously (Kramer et al., 2014). Hierarchical cluster analysis and visualization of proteins enriched in various function categories were performed using *gplots* and *ggplot2* packages in R Bioconductor.

## Results

### Summary of Dataset Generated from Proteomics Analysis

**FIGURE 1:**
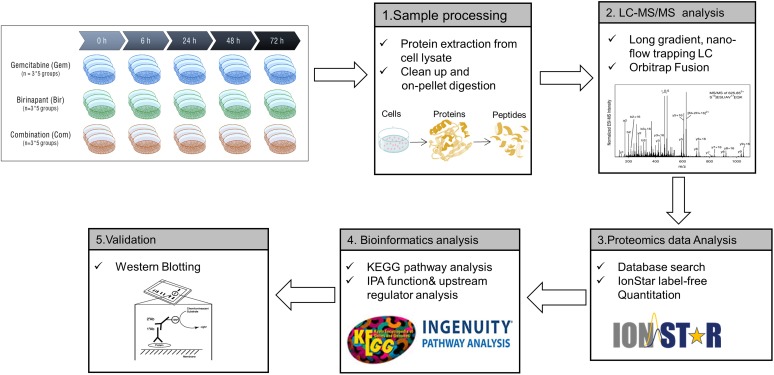
Work flow of the proteomic analysis.

**FIGURE 2:**
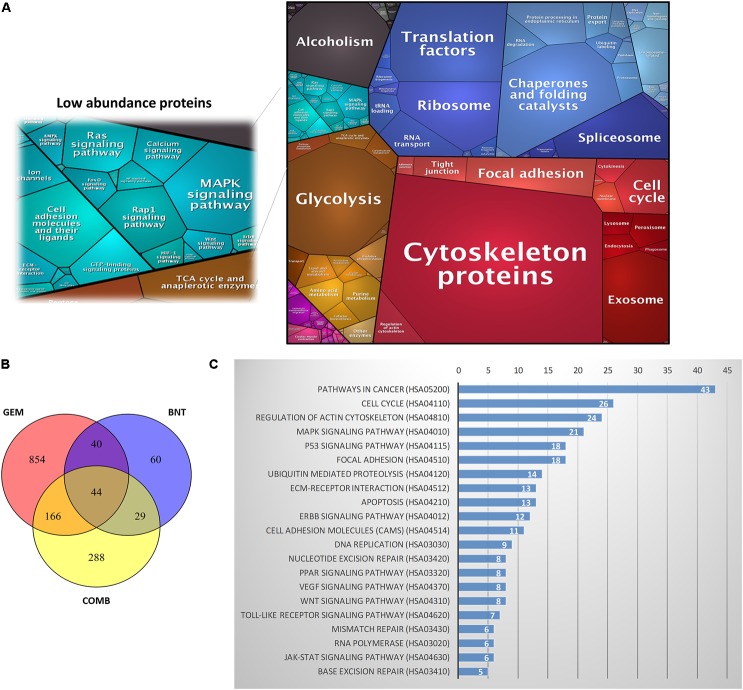
Summary dataset generated from proteomic analysis. **(A)** Proteomap of the proteins identified in PANC-1 cells. **(B)** Distribution of the 1481 proteins that were changed significantly (>1.4-fold and *p* < 0.05) in the three treatment groups: gemcitabine (GEM), birinapant (BNT), and GEM/BNT combined (COMB). A total of 854 proteins were changed only by GEM, 60 were changed only by BNT, and 288 were changed only by the combination (COMB group). Forty-four proteins were changed significantly in all three groups. **(C)** Representative functional pathways containing the largest number of identified proteins altered by the treatments; the number of proteins identified in each pathway is shown in each row.

The PANC-1 cells were treated with gemcitabine (20 nM), birinapant (100 nM) or the two drugs together with the concentrations selected based on the IC_50_ and SC_50_ values from previous cell culture studies (Zhu et al., 2015). Protein samples were collected for LC-MS-based proteomics analysis at five time points (0, 6, 24, 48, and 72 h) with three biological replicates per point. The proteomics workflow (**Figure 1**) consisted of sample processing methodologies, reproducible and in-depth LC-MS analyses, and data analysis workflows that provided extensive proteome coverage and high accuracy and precision. Technical details of this workflow have been published (Elias et al., 2005; Sadygov et al., 2006; Huang et al., 2009; Searle, 2010; Kanehisa et al., 2012; Kim and Pevzner, 2014; Kramer et al., 2014; Nouri-Nigjeh et al., 2014; An et al., 2015; Shen et al., 2015, 2017). A total of 4069 proteins were identified and quantified in the data set consisting of three treatment groups from a total of 45 samples (Supplementary Table S2). These proteins were summarized into a proteomap (Liebermeister et al., 2014) to visualize the composition of proteomes in terms of protein abundance and function (**Figure 2A**). Each protein is represented by a polygon, and the area of each polygon reflects the protein abundance. Functionally related proteins appear in adjacent regions. At the broadest level, the cytoskeleton proteins and proteins related to glycolysis dominated. Cell cycle proteins, which are usually in low abundance in normal cells, showed appreciable abundance in PANC-1 cells, reflecting the active proliferating status of these cells. Most proteins related to cell signaling, such as MAPK or Ras, were classified as low-abundance, although they are functionally important. The low abundance of these important proteins created technical challenges for accurate quantification.

The ANOVA analysis revealed that changes in 1481 proteins in the treatment groups were statistically significant, and the distribution of the number of changed proteins is summarized in **Figure 2B**. Using DAVID, with a focus on the KEGG database, the identified proteins were clustered into 157 pathways, and approximately 35 appeared to be functionally relevant to cancer progression, including cell cycle, MAPK, p53, apoptosis, and DNA replication pathways. Representative pathways are shown in **Figure 2C**. In order to represent the magnitude of protein changes directly on the signaling pathway maps generated from the KEGG database, the fold-change of each protein was log-transformed, and the area between the baseline and the effect curve was calculated. The magnitude of the drug effect on each identified protein is represented by different colors and intensities (Supplementary Figure S1). By this means, protein changes were visualized directly on the plots of signaling pathways (Supplementary Figures S2–S4).

### Exploration of Mechanisms of Drug Action and Interactions

#### Beneficial Interactions from Cell Cycle Regulation and DNA Damage Responses

**FIGURE 3:**
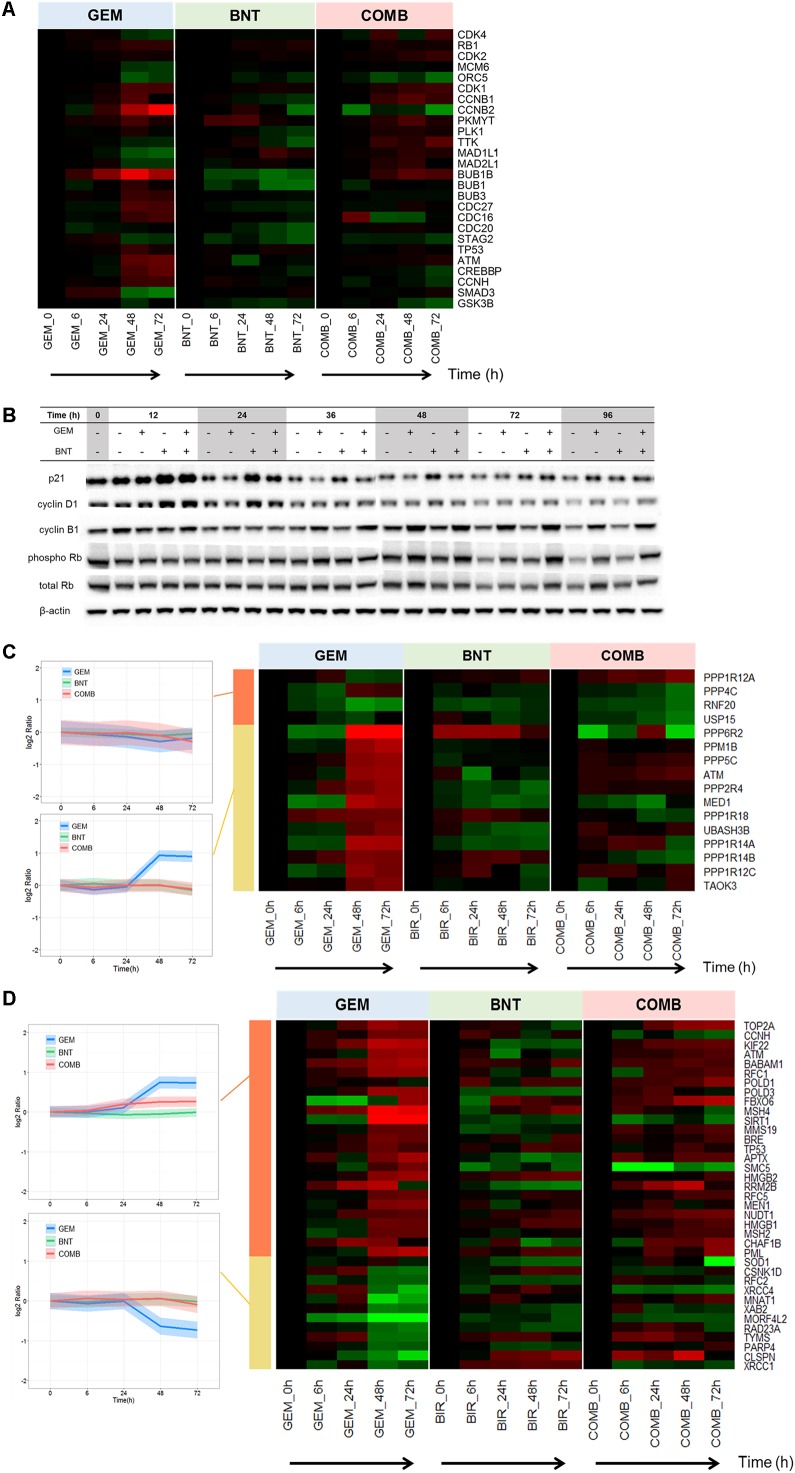
Pathways regulating cell cycle progression, DNA damage responses, and DNA repair affected by gemcitabine/birinapant treatment. **(A)** Heat map representing the time-dependent changes in the abundance of specific proteins related to cell cycle regulation. **(B)** Western blot validation of key cell cycle regulation proteins. Heat map representing the time course and magnitude of change in proteins related to **(C)** regulation of DNA damage responses by the phosphatase and ubiquitin-proteasome system, and **(D)** DNA repair. Proteins in **(C,D)** were further clustered according to different temporal patterns in response to gemcitabine vs. the combined drugs by *k-means clustering* in R Bioconductor. In the heat map plots, *red* indicates up-regulation and *green* indicates down-regulation. The intensity of the color reflects the fold-change of the protein. The western blots **(B)** for 0, 12, 24, and 36 h were from one membrane, and the blots from 0, 48, 72, and 96 h were from a second membrane; the intensity of the blot images was adjusted so that the bands from the two membranes for time 0 h and the loading controls were the same intensity. The proteins relevant to each gene name in the heat map are summarized in Supplementary Table S1.

From the signaling plots, proteins regulating all cell cycle phases were altered in the treatment groups. The proteins related to cell cycle progression were perturbed most extensively by gemcitabine, whereas birinapant showed relatively milder effects (Supplementary Figures S2, S3). Treatment-mediated changes in key regulating proteins (e.g., cyclin B1, pRb, p21) were confirmed by western blot analysis (**Figure 3B**). We investigated reasons for the observed changes in these proteins, particularly in light of previously observed effects of these drugs on cell cycle distributions (Zhu et al., 2015). Gemcitabine can arrest DNA synthesis directly through its incorporation into DNA and the depletion of deoxynucleotide triphosphate pools (Morgan et al., 2005). Such replication stress can activate DNA damage responses (DDR), further activate ATM/Chk2 and ATR/Chk1 check point proteins (Dai and Grant, 2010), and alter diverse downstream effectors regulating cell cycle progression (e.g., cdc25, cyclins, CDKs, BUBs) (**Figure 3A**). Birinapant activated cyclins and CDKs slightly and p21 protein strongly (**Figures 3A,B**), and such changes may be mediated by NF-κB (Karin et al., 2002). The target of birinapant, cIAPs, could also interact directly with E2F1 in all stages of the cell cycle (Cartier et al., 2011), and potentially mediated the altered cell cycle regulators.

Various factors such as endogenous/metabolic (e.g., reactive oxygen species, stalled replication forks) or environmental (e.g., UV, ionizing radiation, genotoxic agents) effects cause DDR (Dai and Grant, 2010). The DDR represent a network of signaling that involves multiple pathways, and coordinates both pro-survival mechanisms such as cell cycle arrest and DNA repair, and pro-death mechanisms such as apoptosis. The balance between the two fates can be controlled by thresholds (abundance of negative regulators) in different pathways (Roos et al., 2015). Proteins involved in DDR are regulated by protein phosphatases (PPs) and the ubiquitin-proteasome system (Mu et al., 2007; Freeman and Monteiro, 2010). We observed that gemcitabine induced significant changes in these PPs and ubiquitin-proteasome proteins (**Figure 3C**) and in proteins involved directly in DNA repair (**Figure 3D**). These protein changes would contribute to cell survival and gemcitabine resistance. The changes in DDR regulation and DNA repair elicited by gemcitabine were diminished in the presence of birinapant (**Figures 3C,D**), and this reduction in the survival and resistance responses elicited by gemcitabine represents one of the major beneficial mechanisms of this combination. The specific mechanism underlying this effect of birinapant is unclear (Ge et al., 2015; Roos et al., 2015).

Overall, the cell cycle regulators were significantly changed in the drug combination group, and the effects mostly appeared to resemble the pattern of responses to gemcitabine alone. This finding demonstrates that gemcitabine-mediated cell cycle arrest is maintained for the two-drug combination. The major beneficial mechanism is the down-regulation by birinapant of gemcitabine-mediated activation of DDR regulation and DNA repair. The drug combination effects at the proteome level are consistent with the enhanced S-phase arrest by combined gemcitabine/birinapant, compared to gemcitabine alone, that we observed previously (Zhu et al., 2015).

#### Alterations in the MAPK-p38 and NF-κB Pathways

**FIGURE 4:**
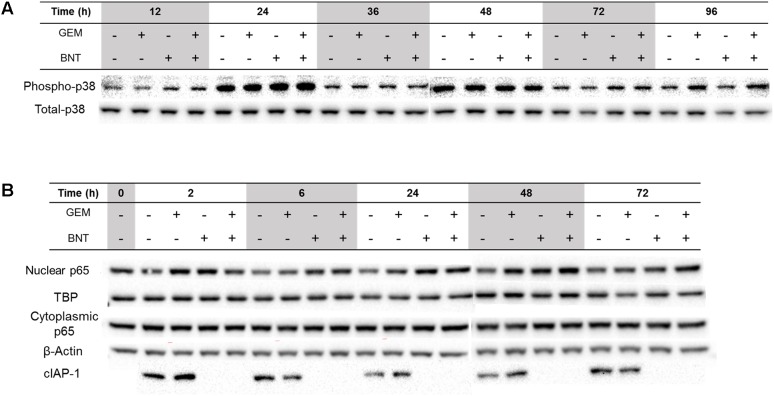
Proteins in the MAPK-p38 and NF-κB affected by gemcitabine and birinapant, alone and in combination. Pathways with significantly changed proteins are shown for **(A)** MAPK-p38 and **(B)** NF-κB.

Disturbance of the MAPK-p38 signaling pathway was observed with both gemcitabine and the combination, but was perturbed only slightly by birinapant alone (Supplementary Figure S3). The MAPK-p38 pathway may be activated by gemcitabine-induced DDR mediated *via* the protein TAO (Raman et al., 2007), and contribute to the caspase-8-mediated apoptosis pathway (Nakashima et al., 2011). This pathway may also be activated by TNF-α *via* TAK1 (Dai et al., 2012), or by FasL through ASK1 (Farley et al., 2006). Despite various upstream stimuli activated by the two drugs in the MAPK pathway, western blot analysis revealed that gemcitabine induced only a slight increase in the downstream p38 phosphorylation, and the effect of birinapant alone on p38 was slight and limited to early time points (**Figure 4A**). This finding is probable evidence of activated negative regulators of the MAPK pathway, such as PP2CB and PP5, the PPs regulating DDR (Morita et al., 2001; Dai et al., 2012).

The activation of NF-κB by gemcitabine and birinapant as single agents was observed, as revealed by the increased abundance of nuclear p65 (normalized by protein TBP) compared to the unchanged cytoplasmic p65 (normalized by protein β-Actin) by western blots in **Figure 4B**. Gemcitabine-mediated activation of NF-κB is part of the DDR, and is considered to be a cell survival pathway contributing to gemcitabine resistance (Voutsadakis, 2011). Literature reports indicate that activation could be mediated by NF-κB essential modulator or TAK1 (Jin et al., 2009; McCool and Miyamoto, 2012). Birinapant can interrupt the NF-κB pathway by degradation of TRAF2-bound cIAP1 (Benetatos et al., 2014) and cIAP2 (**Figure 4B**). In previous studies, after pretreatment with TNF-α, birinapant blocked TNF-α-mediated activation of NF-κB, which requires the formation of a complex of cIAP-bound TRAF2 with TNFR and TRADD. The degradation of cIAPs also switches TNF-α/NF-κB signaling to the formation of a RIPK1:caspase-8 protein complex and increases caspase-8 activation (Benetatos et al., 2014). In our studies, which lack pretreatment with TNF-α, we observed activation of NF-κB by birinapant at early time points and slight, oscillating induction of activity at later times (**Figure 4B**). It is possible that rapid birinapant-mediated degradation of cIAPs leads to rapid recruitment of RIP1 to TNF-R1 and subsequent p65 phosphorylation (Vince et al., 2007). Increased NF-κB activity would be expected to induce secretion of TNF-α in a positive-feedback manner, but the positive loop is interrupted and switched to caspase-8 signaling in the presence of birinapant (Benetatos et al., 2014). This could explain why NF-κB was activated strongly in the combination group. Despite the fact that NF-κB contributes to gemcitabine resistance, the addition of birinapant to gemcitabine partially shifts the NF-κB signaling toward caspase-mediated apoptosis.

#### Beneficial Interactions in the Apoptosis Pathway

**FIGURE 5:**
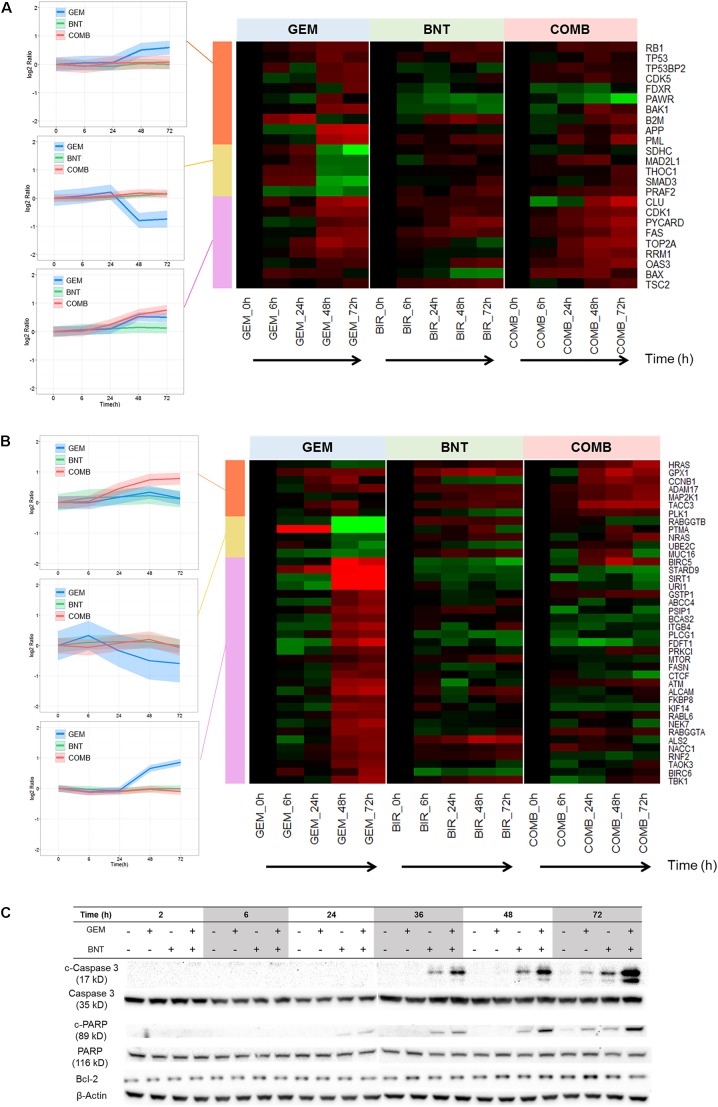
Pathways related to apoptosis affected by gemcitabine/birinapant treatment. Significantly altered proteins involved in **(A)** pro-apoptotic and **(B)** anti-apoptotic responses that were quantified by proteomic analysis are summarized in the heat map plot. **(C)** Western blot analysis validating changes in key apoptosis-related proteins. Clustering of proteins and colors are defined in the **Figure 3** legend. The proteins relevant to each gene name in the heat map are summarized in Supplementary Table S1.

Apoptosis pathways are well-characterized in the literature. Our data show that pro-apoptotic proteins were activated in all three treatment groups, and enhanced slightly in the combination group (**Figure 5A**). The majority of anti-apoptotic proteins were induced by gemcitabine, which would contribute to drug resistance; this induction was countered by the addition of birinapant (**Figure 5B**).

We explored possible mechanisms to rationalize the observed protein-level changes. Both intrinsic and extrinsic apoptosis may be induced by DDR mediated by p53, c-Myc, and TAO. Gemcitabine-mediated strong up-regulation of Bax (**Figure 5A**) and slight induction of Bcl-2 (**Figure 5C**), indicating potential activation of intrinsic apoptosis. The extrinsic apoptosis pathway is also activated. Gemcitabine has been reported to induce Fas ligand expression, which could be mediated by down-regulated miR-21 expression or increased JNK phosphorylation and activated protein 1 (AP-1) activity (Wang et al., 2013; Roos et al., 2015). Fas ligation induced by gemcitabine can further activate the MAPK-p38 pathway *via* ASK1 (Morita et al., 2001), and lead to caspase-8-mediated extrinsic apoptosis.

Birinapant also exerted a range of modulating effects upon apoptosis. As mentioned above, it can switch the TNF-α/NF-κB signaling toward caspase-8-mediated extrinsic apoptosis. Birinapant also promotes apoptosis by binding to and antagonizing XIAP (IAP proteins X chromosome-linked IAP) and ML-IAP (melanoma IAP), which block apoptosis by suppressing the activity of caspases (Fulda and Vucic, 2012). Therefore, strong pro-apoptotic signaling results from the combination of birinapant with gemcitabine, as confirmed by induced cleaved PARP and caspase-3 (**Figure 5C**), which confirms conclusions from the proteomic analysis.

#### Potential Beneficial Drug Interactions in Cell Migration

**FIGURE 6:**
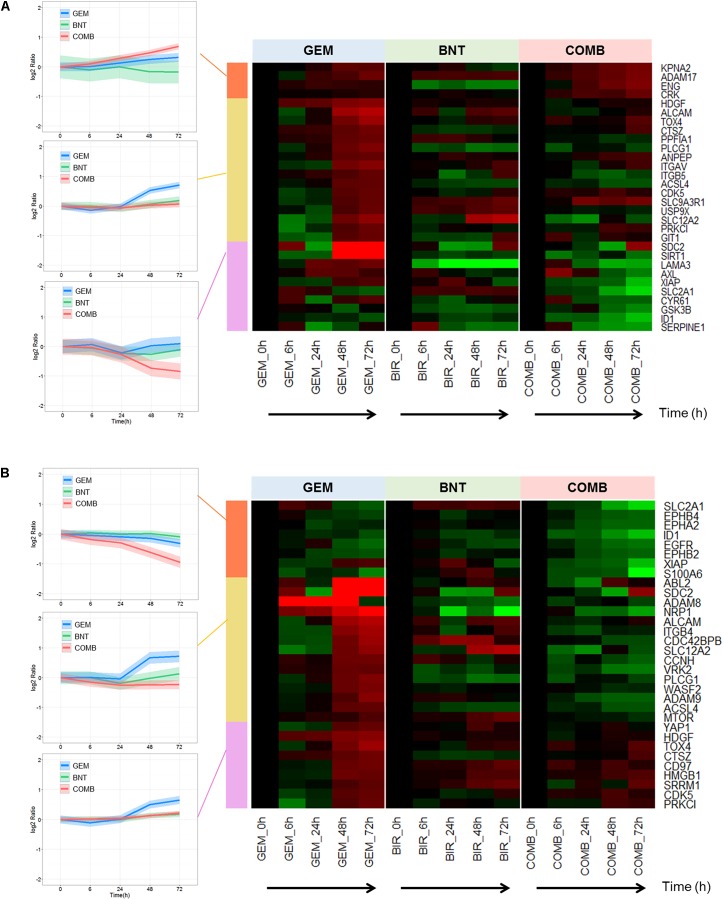
Proteins involved in migration and invasion affected by gemcitabine/birinapant treatment. Significantly altered proteins related to **(A)** induction of migration and **(B)** increasing invasion that were identified and quantified by proteomic analysis and summarized in the heat map plot. Clustering of protein and colors are defined in the **Figure 3** legend. The proteins relevant to each gene name in the heat map are summarized in Supplementary Table S1.

Numerous proteins promoting cell migration and invasion were up-regulated by gemcitabine, but many of those changes were reversed by the addition of birinapant to gemcitabine (**Figure 6**). Potential underlying mechanisms in this pathway were investigated. Actin polymerization and disassembly are related to cellular motility, and Abi2, wave2, and Arp2/3 regulate directly the formation of filopodia and lamellipodia at the leading edge (Supplementary Figure S4). Co-expression of Arp2 and wave2 is correlated with poorer patient outcomes, and may be involved mechanistically in cancer metastasis (Otsubo et al., 2004; Semba et al., 2006). We observed increased expression of these proteins after gemcitabine exposure, and this induction was blocked by birinapant (Supplementary Figure S4). Previous *in vitro* studies confirmed that gemcitabine-treated cells showed increased unidirectional migration and expression of epithelial–mesenchymal transition markers (Quint et al., 2012). Based on the observed data, birinapant appears to reduce the potential for cancer metastasis that is promoted by gemcitabine.

### Application: Exploration of Mechanisms Related to Gemcitabine Resistance

We sought to identify potential applications of the information derived from the proteomic analysis. Our previous analysis identified several signaling pathways relevant to gemcitabine resistance: regulators of DDR, DNA repair proteins, anti-apoptosis proteins, and pro-migration/invasion proteins. Proteins induced by gemcitabine in these pathways merit further investigation, as antagonizing those proteins could be beneficial to reverse drug resistance. The proteins summarized in the heat maps (**Figures 3C,D, 5B, 6**) were analyzed by *k-means clustering* in R Bioconductor to identify different temporal patterns of response to gemcitabine vs. the combination. Within the gemcitabine-induced proteins, those down-regulated in the combination group may be responsible for the beneficial effects of the drug combination (as described above), and those unaffected by birinapant represent potential targets to enhance the efficacy of current gemcitabine-based combination therapy further.

In the anti-apoptotic protein group, the majority of significantly changed proteins were up-regulated by gemcitabine, including proteins of the IAP family (BIRC5/survivin, BIRC6/Bruce) and the E3 ubiquitin ligase class (Ring2) with similar functions as IAP (**Figure 5B**). This confirmed the IAP family as an important contributor to gemcitabine resistance and a good drug target candidate. Similarly, gemcitabine-induced proteins histone deacetylase (SIRT1), farnesyltransferase (FDFT1), and proteins that control cell proliferation, such as mTOR, Ras (HRAS), and pre-mRNA-splicing factor (BCAS2) also represent targets to improve gemcitabine efficacy and reduce chemoresistance (**Figure 5B**). Of these gemcitabine-induced anti-apoptotic proteins, approximately 70% were down-regulated by the addition of birinapant. However, six proteins remained slightly up-regulated in the combination group, including transforming acidic coiled-coil protein 3 (TACC3; **Figure 5B**), and these proteins represent potential targets to enhance the efficacy of current gemcitabine/birinapant combinations further.

Proteins involved in pro-migration or invasion were also investigated. Several gemcitabine-induced proteins stood out because of their large change (>10-fold) in abundance, such as the A disintegrin and metalloproteinases (ADAM8, ADAM17), neuropilin-1 (NRP1), and insulin-like growth factor binding proteins (SDC2, CYR61) (**Figure 6**). Others were induced to a lesser degree, but are better studied and more clearly associated with migration and metastasis, including integrins (ITGAV, ITGB5, ITGB4; Desgrosellier and Cheresh, 2010), and cell adhesion molecules (ALCAM; Tachezy et al., 2012). Interestingly, most of these promising targets to reduce cell migration were down-regulated with addition of birinapant to gemcitabine.

Proteins responsible for DDR regulation, especially the protein serine/threonine phosphatases (PSTPs; **Figure 3C**), may also constitute promising targets to reverse gemcitabine resistance *via* enhancement of p38-mediated apoptosis and impairment of DNA repair. Proteins directly responsible for DNA repair (DNA mismatch repair proteins, DNA polymerases, XRCCs, Replication factor C; **Figure 3D**) could be targeted to enhance the anti-proliferative effect mediated by gemcitabine-stalled replication.

### Application: Upstream Regulator Analysis

**FIGURE 7:**
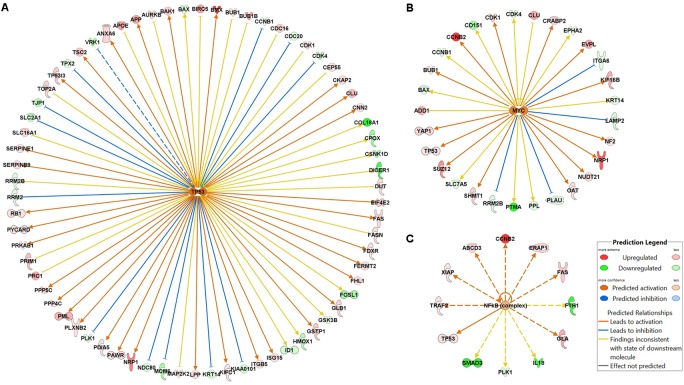
Potential gemcitabine/birinapant effects on upstream regulatory proteins. Selected upstream regulator analysis was performed using Ingenuity Pathway Analysis for **(A)** p53, **(B)** Myc, and **(C)** NF-κB. The figure box defines the connections and colors used to signify up- and down-regulation.

The roles of key and high-level regulators in the drug response pathways can also be explored with this comprehensive proteomic analysis. The IPA URA can elucidate upstream transcriptional regulators based on the observed gene/protein expression changes *via* causal analysis approaches, and predict whether such regulators are activated or inhibited based upon the pattern of up- or down-stream gene changes (Kramer et al., 2014). We applied URA to investigate the critical upstream regulators of the identified signaling pathways altered by drug exposure. These upstream regulators included NF-κB, p53, and c-Myc. P53 was identified as activated by gemcitabine in URA (**Figure 7A**) and confirmed by experimental quantification (**Figure 3A**). It was mapped with approximately 70 downstream proteins relating to such functions as cancer cell proliferation, apoptosis, cytoskeleton, and migration. For example, p53 is involved in the activation of DDR by up-regulating serine/threonine phosphatases PPP4C and PPP5C (**Figure 7A**), contributing to cell survival (Freeman and Monteiro, 2010), and it activates tyrosine kinase BMX (**Figure 7A**) and promotes cell proliferation (Holopainen et al., 2012); however, p53 also suppresses cell cycle progression by up-regulating BUB proteins, inhibits the expression of cancer markers such as COL18A1 and DICER1, and induces FAS expression to promote apoptosis (**Figure 7A**). URA also predicted that c-Myc would be activated by gemcitabine, and the impact on the downstream pathways would be extensive and diverse (**Figure 7B**). For example, the growth-promoting oncoprotein PTMA is down-regulated by c-Myc, whereas NRP1, a co-receptor for VEGF and TGF-β that contributes to cell proliferation and migration, is up-regulated by c-Myc. The NF-κB was identified as also activated by gemcitabine through URA (**Figure 7C**), and this prediction was confirmed by western blot analysis (**Figure 4B**). NF-κB induced anti-apoptotic proteins such as IAP or Bcl-2, and also induced overexpression of cyclin B related to arrest in cell cycle progression (**Figure 7C**). Overall, these key upstream regulators usually control multiple downstream pathways and carry out complex roles in functions related to cell progression or repression. As a result of their extensive functions, these upstream regulators may not be good candidates for direct targeting.

## Discussion

This study extends our previous investigation of the mechanisms of gemcitabine and birinapant actions and interactions. Here, comprehensive proteomic analysis provided a broader perspective and accurate high-throughput quantification of inter-related signaling pathways. Information from western blot analysis and a literature search confirmed and extended the proteomic observations to provide more detailed insights. The mechanisms of drug action were characterized at a proteomic level in greater detail than possible previously. We conclude that the positive effects of the gemcitabine/birinapant combination arise from several effects: (1) the pro-survival DDR and DNA repair induced by gemcitabine was dampened by birinapant; (2) apoptosis signaling was enhanced for the combination; and (3) gemcitabine-induced effects upon cell migration/invasion pathways were reduced by birinapant. These signaling networks are also related to gemcitabine chemoresistance, and important proteins in these pathways could be candidates for therapeutic targeting.

Particularly, SIRT1 was up-regulated by gemcitabine and involved in both anti-apoptotic and pro-invasion pathways. Various preclinical studies demonstrate SIRT1 as an effective anti-cancer target (Jung-Hynes et al., 2009). Combining SIRT1 inhibitors with 5-fluorouracil showed synergistic effects in inhibiting tumor growth and metastasis in breast cancer cells (Hwang et al., 2014). In addition, miR-34a was found to be a natural SIRT suppressor (Bellio et al., 2016), therefore, safely inducing the expression of miR-34a can be a promising approach. TACC3 is an important spindle-regulatory protein in the centrosome-microtubule network during mitosis, and remained to be up-regulated after treatment of combination. Overexpression of TACC3 has been shown in a variety of human cancers, and correlated with lower survival rate and tumorigenesis (Yun et al., 2015; Sun et al., 2017). Knockdown of TACC3 resulted in enhanced efficacy of other cytotoxic agents (e.g., paclitaxel) in preclinical models (Yim et al., 2009). Therefore, reducing TACC3 expression has the potential to introduce additional benefit after combining agents inhibiting anti-apoptotic proteins and agents that cause DNA stress. PSTPs, including subunits of PPP1, 2, 5, and 6 were all significantly up-regulated after gemcitabine treatment. This protein family is highly regulated and target specific, which renders them as potential good drug targets. Several natural compounds with immunosuppressive properties can specifically bind to and inhibit catalytic subunits of PSTPs (e.g., cyclosporin A, FK506, cantharidin, and fostriecin; McConnell and Wadzinski, 2009). Cyclosporin A and cantharidin has been shown in preclinical studies to synergize with cytotoxic agents (Jeske et al., 2003; Wang et al., 2015). However, these agents also exert significant toxicity, and optimization of the safety profile of PSTP inhibitors will be the major challenge. Clinical studies to inhibit DNA repair processes also have been initiated. However, several challenges are faced: due to the pathway crosstalk and functional redundancy, it is hard to disrupt DNA repair through one target, and a combination approach is more feasible. In addition, the nature of the target of the DNA repair proteins makes it difficult to develop specific inhibitors. Therefore, although the concept is valid, it is challenging to find a specific and efficient way to interrupt the DNA repair process (Kelley et al., 2014).

This proteomics approach provides advantages in its unbiased view of cellular signaling pathways and response networks. Simultaneous quantification of large numbers of diverse protein targets facilitates the evaluation of drug response mechanisms. Proteomic analysis can also assist in selecting representative proteins that can reveal the status of the network, or representatives of a protein class that reflect the status of proteins with similar functions. A unique feature of this study is the multi-dimensional nature of the information obtained: the three drug treatments (gemcitabine, birinapant, or combination) serve as different perturbations of the biological system, and the time series analysis places the results of the perturbations in a temporal context that can suggest sequential processes. Therefore, this approach has even greater potential impact if combined with transcriptional profiling, phenotypes in drug-treated cells, and mathematical models that assist in identifying quantitative relationships and providing therapeutic insights (Lee et al., 2012).

The major limitation of the study is that the changes of protein abundance after treatments were normalized by the protein abundance at time 0, instead of by the control group at each time point. The instrument capacity limited the sample size in each batch; therefore the control group was analyzed in a separate batch. Only 85% of the proteins could be identified in the control compared to the treatment groups, therefore normalizing the treatment groups by control group would diminish the number of proteins identified. Analysis of the control group showed that only less than 2% of proteins at each time point met the criteria of significant change (Supplementary Figure S5), indicating a consistency over time at the macroscale. However, further investigation of individual proteins may require consideration of changing baselines. Other limitations in the study exist. First, although observed changes in the abundance of specific proteins implicates strong pro-migration effects of gemcitabine, which appeared to be mitigated by combination with birinapant, this potentially important finding requires further biological and functional validation. Second, of the large number of proteins in various pathways altered by the combination, it was only possible to investigate a limited selection in detail and to validate them by experiments and literature search. Additional functional pathways, such as metabolism, insulin signaling pathways, toll-like receptor pathways may also require exploration in detail as they hold the potential to yield important insights into therapeutic interventions for pancreatic cancer.

## Author Contributions

The manuscript was written through contributions of all authors. XZ and XS performed the experiments, analyzed the data, and wrote the first draft of manuscript. JQ, RS, and WJ supervised the students, directed data analysis, and revised the manuscript. All authors have given approval to the final version of the manuscript.

## Conflict of Interest Statement

The authors declare that the research was conducted in the absence of any commercial or financial relationships that could be construed as a potential conflict of interest.

## Footnotes

1 https://github.com/shxm725/IonStarstat
