# Supplementary material for: Proteomic Analysis of Combined Gemcitabine and Birinapant in Pancreatic Cancer Cells
Source: Front Pharmacol. 2018 Feb 19;9:84. doi: 10.3389/fphar.2018.00084 (PMC5827530; doi:10.3389/fphar.2018.00084)
Supplement: Supplementary file 1 [file Presentation_1.pdf]

Figure S-1. Conversion of fold-changes of protein into area under the effect curve (AUEC). *Red* represents up-regulation and *green* indicates down-regulation.

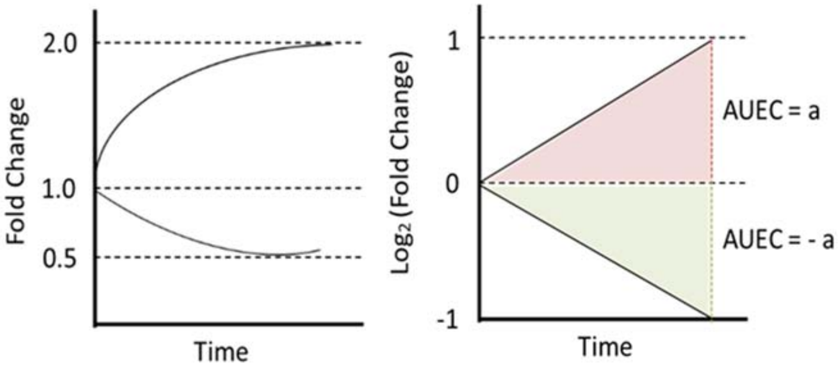

Figure S-2. Effects of gemcitabine and birinapant, alone or combined, on cell cycle regulation pathways. Colored pathway nodes denote proteins that were both identified and changed significantly. In the signaling diagrams, *red* indicates up-regulation and *green* indicates down-regulation. The intensity of the color reflects the fold-change of the protein.

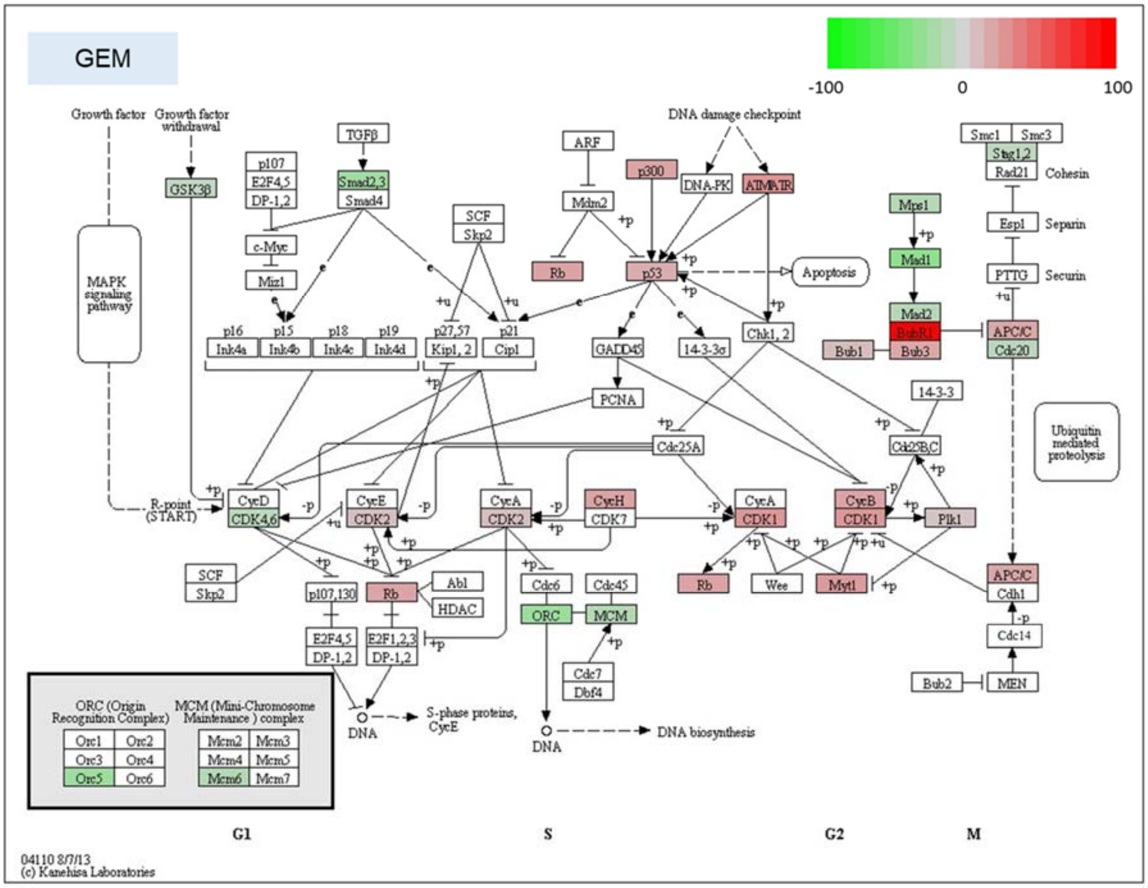

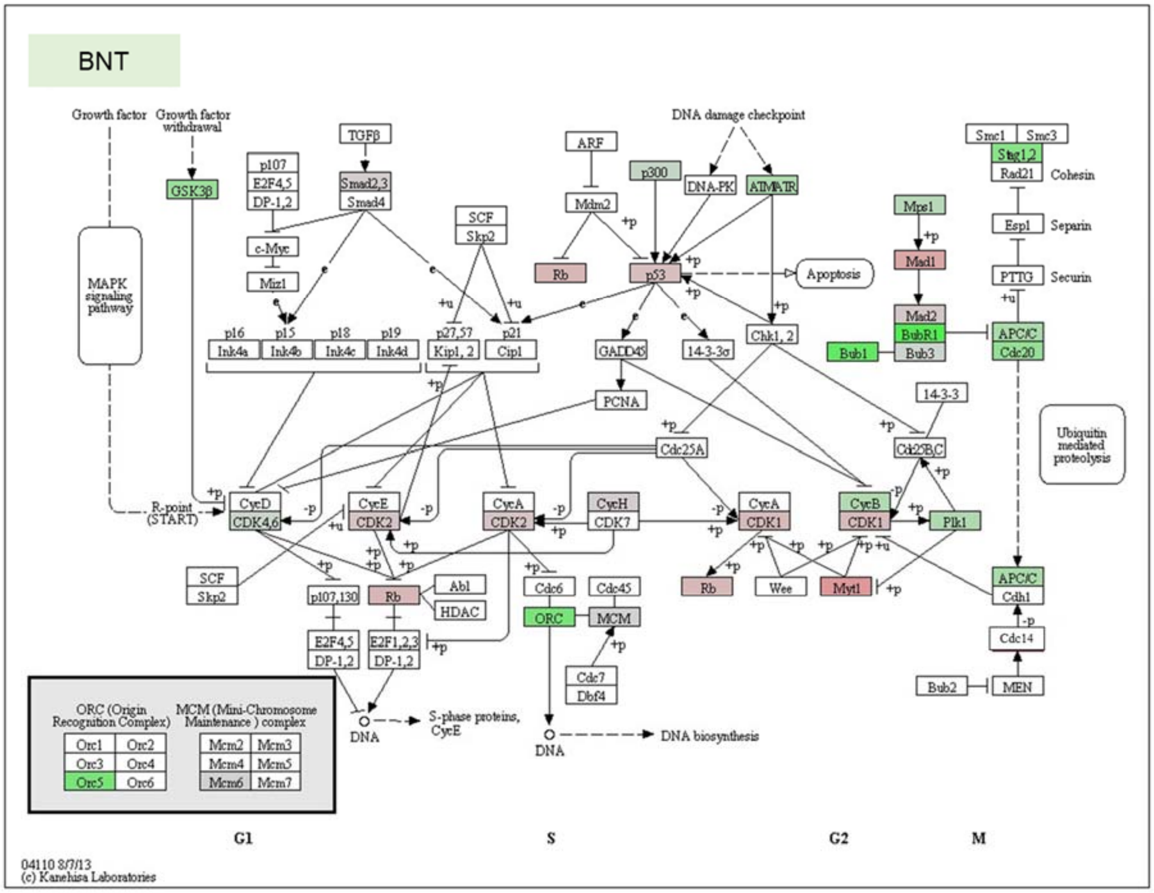
BNT
Growth factor
Growth factor withdrawal
GSK3β
MAPK signaling pathway
p107
E2F4,5
DP-1,2
TGFβ
Smad2,3
Smad4
c-Myc
Miz1
SCF
Skp2
ARF
Mdm2
p300
DNA damage checkpoint
DNA-PK
ATM/ATR
Rb
p53
Apoptosis
p16
p15
p18
p19
Ink4a
Ink4b
Ink4c
Ink4d
p27,57
p21
Kip1, 2
Cip1
GADD45
14-3-3σ
Chk1, 2
PCNA
Cdc25A
CycD
CDK4,6
CycE
CDK2
CycA
CycH
CDK7
CDK1
R-point (START)
p107,130
Abl
HDAC
E2F4,5
DP-1,2
E2F1,2,3
Cdc6
Cdc45
ORC
MCM
Cdc7
Dbf4
S-phase proteins, CycE
DNA
DNA biosynthesis
Wee
Myt1
Smc1
Smc3
Stag1,2
Rad21
Cohesin
Mps1
Esp1
Separin
Mad1
PTTG
Securin
Mad2
BubR1
Bub3
Bub1
APC/C
Cdc20
14-3-3
Cdc25B,C
CycB
Plk1
Ubiquitin mediated proteolysis
Cdh1
Cdc14
Bub2
MEN
ORC (Origin Recognition Complex)
MCM (Mini-Chromosome Maintenance) complex
Orc1
Orc2
Orc3
Orc4
Orc5
Orc6
Mcm2
Mcm3
Mcm4
Mcm5
Mcm6
Mcm7
G1
S
G2
M
04110 8/7/13
(c) Kanehisa Laboratories

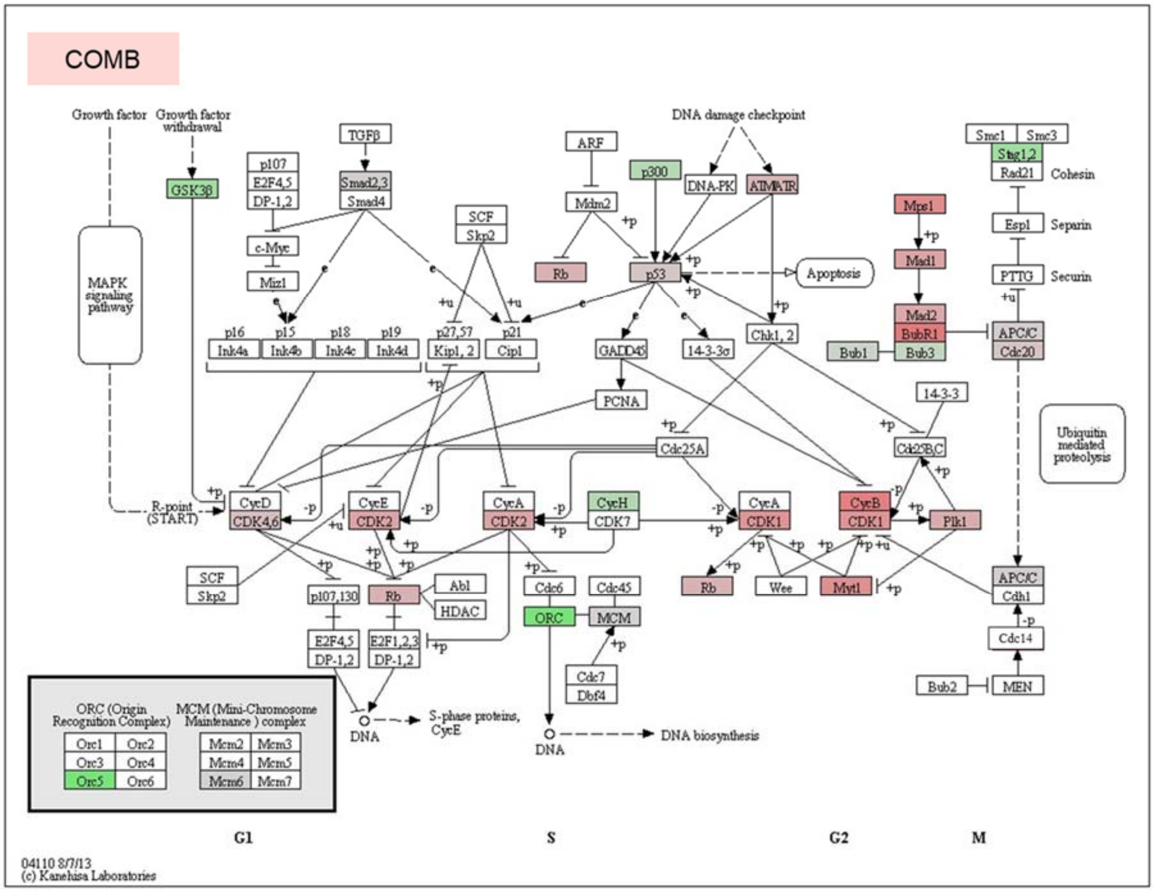

COMB
Growth factor
Growth factor withdrawal
GSK3β
MAPK signaling pathway
R-point (START)
TGFβ
Smad2,3
Smad4
p107
E2F4,5
DP-1,2
c-Myc
Miz1
p16 p15 p18 p19
Ink4a Ink4b Ink4c Ink4d
SCF
Skp2
p27,57 p21
Kip1, 2 Cip1
ARF
Mdm2
p300
Rb
DNA damage checkpoint
DNA-PK
ATM/ATR
p53
Apoptosis
GADD45
14-3-3σ
Chk1, 2
PCNA
Cdc25A
CycD
CDK4,6
CycE
CDK2
CycA
CDK2
CycH
CDK7
CycA
CDK1
CycB
CDK1
Plk1
p107,130
Rb
Abl
HDAC
E2F4,5
DP-1,2
E2F1,2,3
DP-1,2
DNA
S-phase proteins, CycE
Cdc6
ORC
Cdc45
MCM
Cdc7
Dbf4
DNA
DNA biosynthesis
Wee
Myt1
Mps1
Mad1
Mad2
BubR1
Bub1
Bub3
14-3-3
Cdc25B,C
Smc1 Smc3
Stag1,2
Rad21
Cohesin
Espl
Separin
PTTG
Securin
APC/C
Cdc20
Ubiquitin mediated proteolysis
APC/C
Cdh1
Cdc14
Bub2
MEN
ORC (Origin Recognition Complex)
Orc1 Orc2
Orc3 Orc4
Orc5 Orc6
MCM (Mini-Chromosome Maintenance) complex
Mcm2 Mcm3
Mcm4 Mcm5
Mcm6 Mcm7
G1
S
G2
M
04110 8/7/13
(c) Kanehisa Laboratories

Figure S-3. Significantly-changed proteins in the MAPK-p38 pathway. Colors are defined in the Figure S-2 legend.

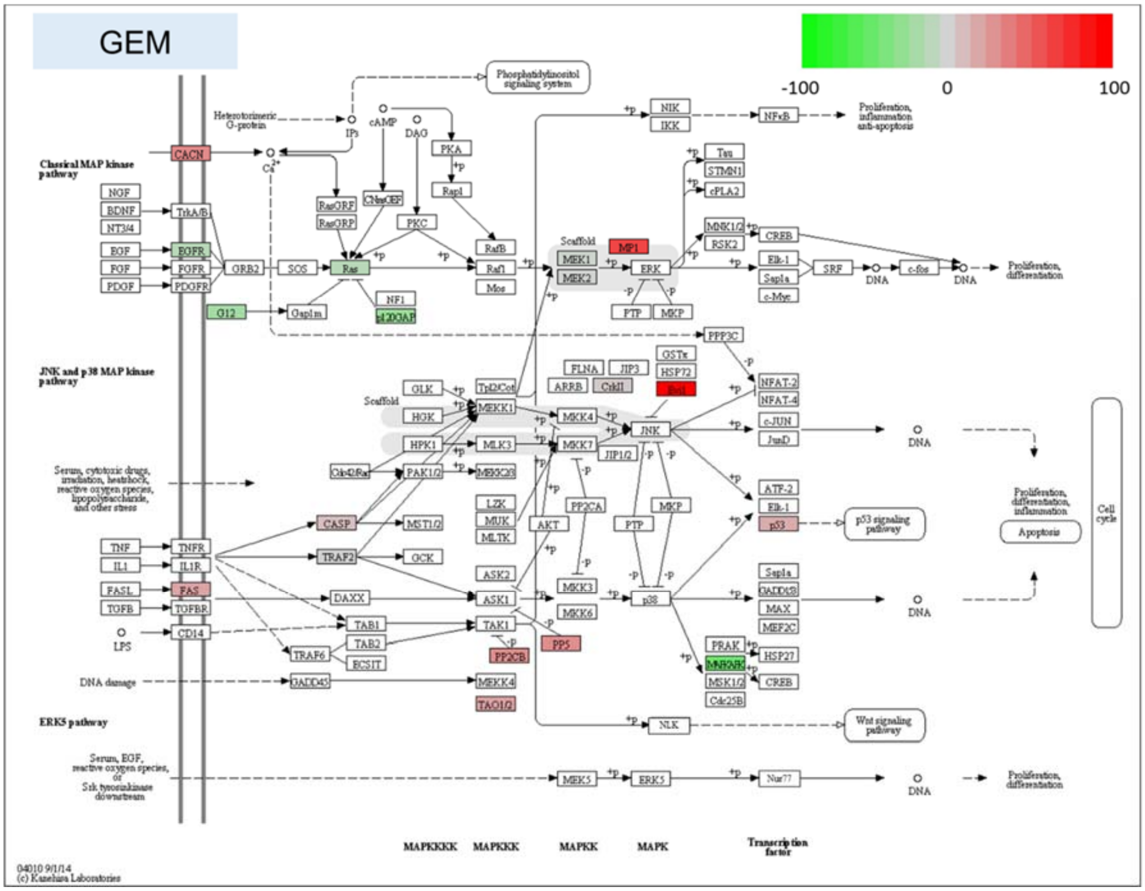

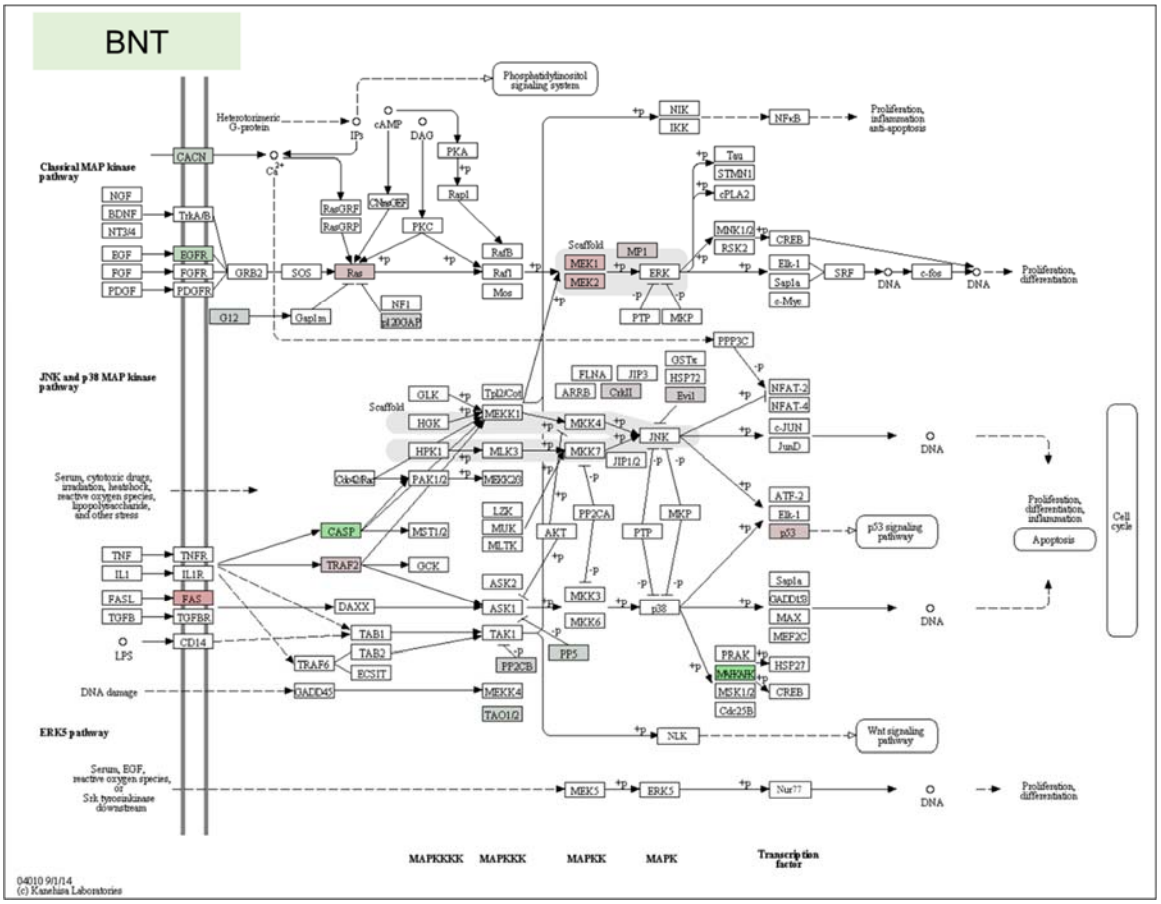
BNT
Classical MAP kinase pathway
JNK and p38 MAP kinase pathway
ERK5 pathway
Phosphatidylinositol signaling system
Proliferation, inflammation anti-apoptosis
Proliferation, differentiation
p53 signaling pathway
Wnt signaling pathway
Apoptosis
Cell cycle
MAPKKKK
MAPKKK
MAPKK
MAPK
Transcription factor
04010 9/1/14
(c) Kanehisa Laboratories

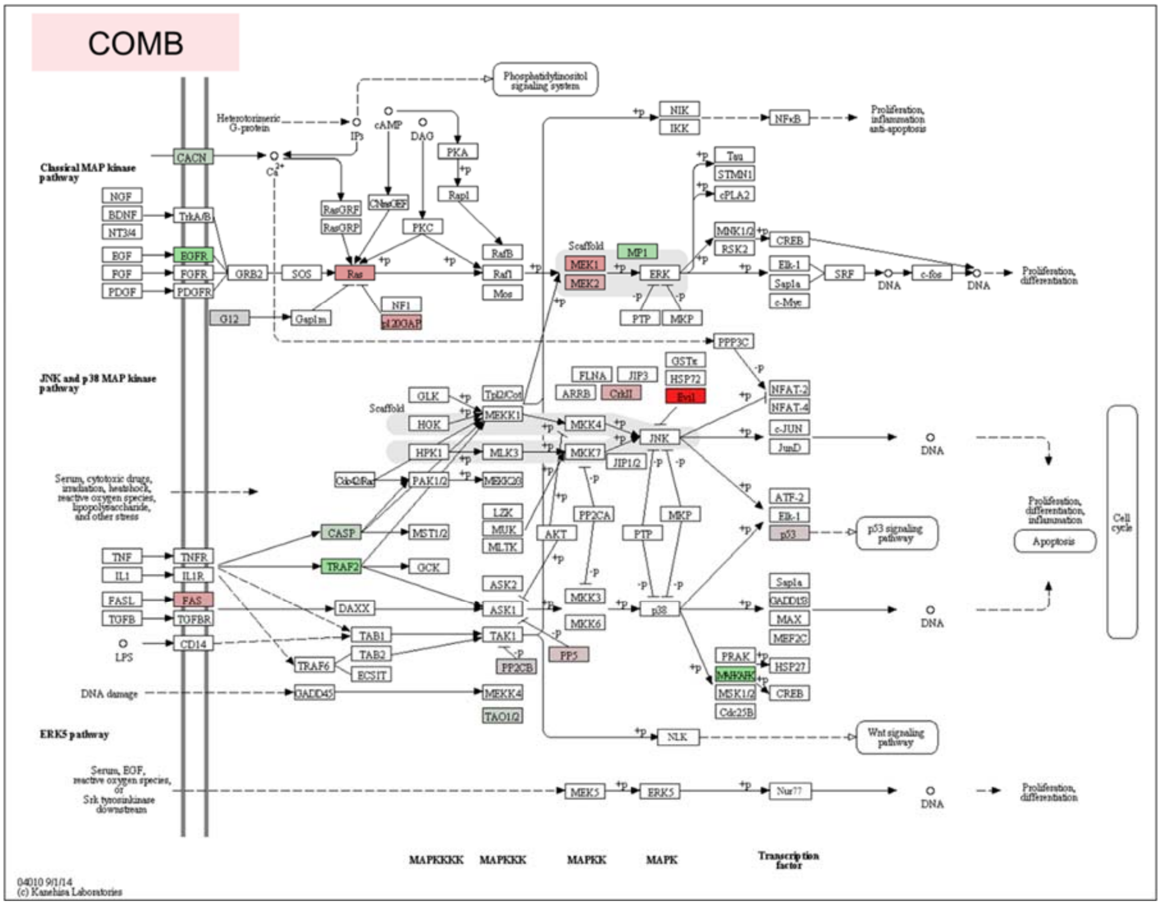
COMB
Classical MAP kinase pathway
JNK and p38 MAP kinase pathway
ERK5 pathway
Phosphatidylinositol signaling system
Heterotrimeric G-protein
CACN
NGF
BDNF
NT3/4
EGF
FGF
PDGF
TrkA/B
EGFR
FGFR
PDGFR
GRB2
SOS
Ras
RasGRF
RasGRP
CNrasGEF
PKC
PKA
Rap1
RafB
Raf1
Mos
NF1
p120GAP
G12
Gap1m
MEK1
MEK2
MP1
ERK
PTP
MKP
NIK
IKK
NFκB
Proliferation, inflammation anti-apoptosis
Tau
STMN1
cPLA2
MNK1/2
RSK2
CREB
Elk-1
Sap1a
c-Myc
SRF
c-fos
DNA
Proliferation, differentiation
PPP3C
NFAT-2
NFAT-4
GLK
HGK
HPK1
Tpl2/Cot
MEKK1
MLK3
MEKK2/3
PAK1/2
Cdc42/Rac
FLNA
JIP3
ARRB
CrkII
GSTπ
HSP72
Evi1
MKK4
MKK7
JNK
JIP1/2
c-JUN
JunD
Serum, cytotoxic drugs, irradiation, heatshock, reactive oxygen species, lipopolysaccharide, and other stress
LZK
MUK
MLTK
AKT
PP2CA
MKP
ATF-2
Elk-1
p53
p53 signaling pathway
Proliferation, differentiation, inflammation
Apoptosis
Cell cycle
TNF
IL1
FASL
TGFB
LPS
TNFR
IL1R
FAS
TGFBR
CD14
CASP
TRAF2
MST1/2
GCK
DAXX
ASK2
ASK1
TAB1
TAB2
TAK1
TRAF6
ECSIT
PP2CB
PP5
MKK3
MKK6
p38
Sap1a
GADD153
MAX
MEF2C
PRAK
MAPKAPK
MSK1/2
Cdc25B
HSP27
CREB
DNA damage
GADD45
MEKK4
TAO1/2
NLK
Wnt signaling pathway
Serum, EGF, reactive oxygen species, or Src tyrosinkinase downstream
MEK5
ERK5
Nur77
MAPKKKK
MAPKKK
MAPKK
MAPK
Transcription factor
04010 9/1/14
(c) Kanehisa Laboratories

Figure S-4. Significantly altered proteins related to regulation of actin cytoskeleton. Clustering of protein and colors are defined in the Figure S-2 legend.

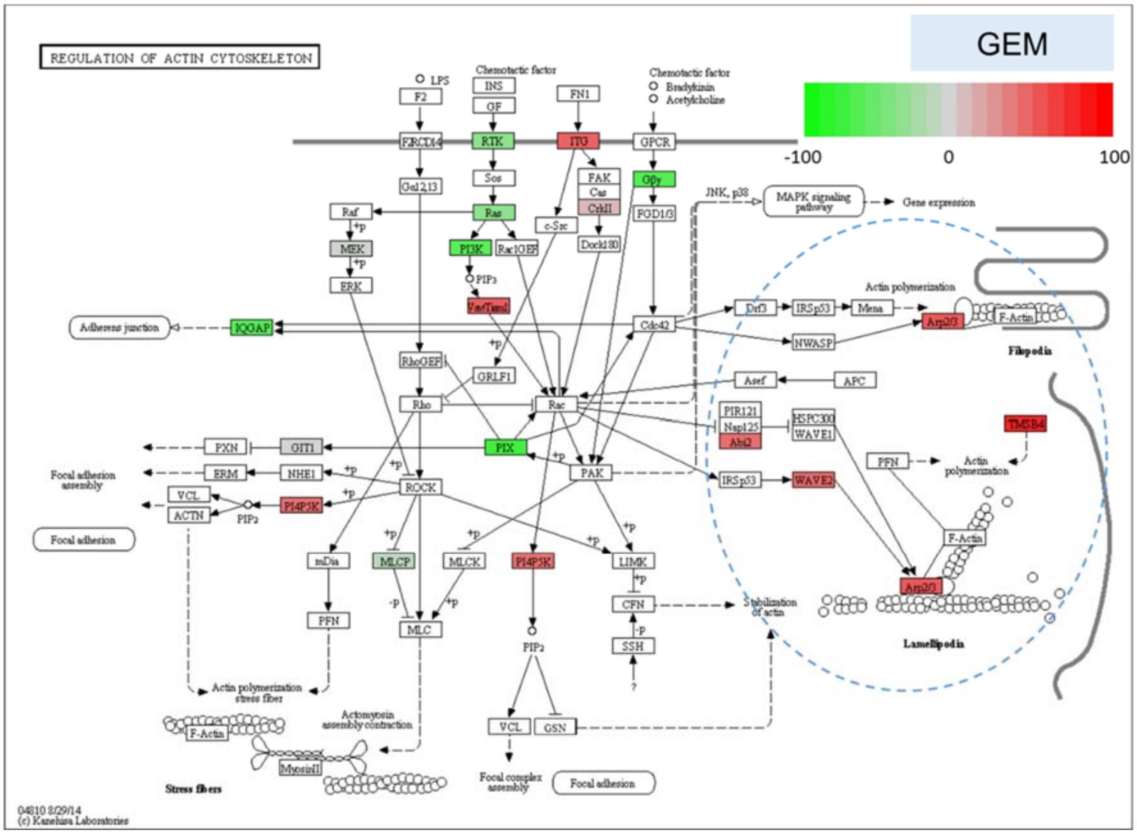

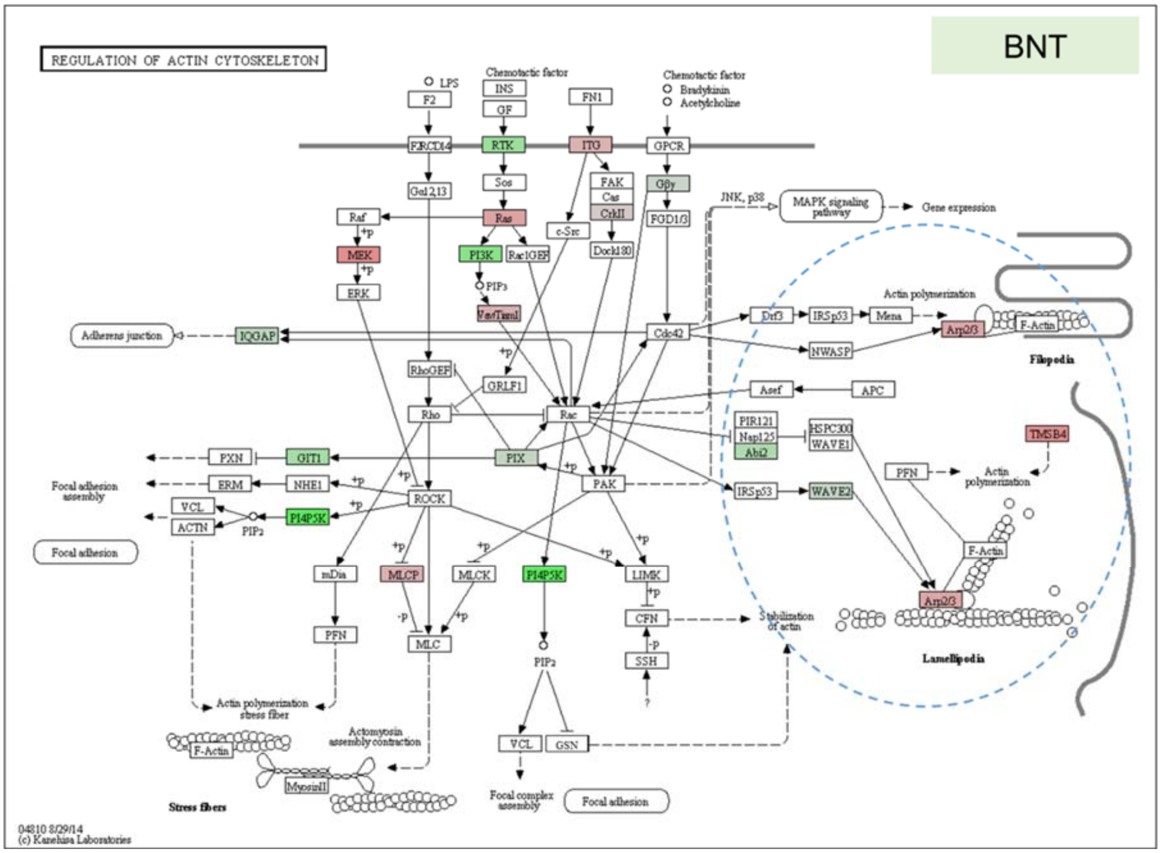
BNT
REGULATION OF ACTIN CYTOSKELETON
LPS
Chemotactic factor
INS
GF
F2
FN1
Chemotactic factor
Bradykinin
Acetylcholine
F2RCD14
RTK
ITG
GPCR
Gα12,13
Sos
FAK
Cas
CrkII
Gβγ
Raf
Ras
c-Src
FGD1/3
JNK, p38
MAPK signaling pathway
Gene expression
MEK
PI3K
Rac1GEF
Dock180
ERK
PIP3
Vav/Tiam1
Actin polymerization
Drf3
IRSp53
Mena
Arp2/3
F-Actin
Adherens junction
IQGAP
Cdc42
NWASP
Filopodia
RhoGEF
GRLF1
Asef
APC
Rho
Rac
PIR121
Nap125
Abi2
HSPC300
WAVE1
TMSB4
PXN
GIT1
PIX
PFN
Actin polymerization
Focal adhesion assembly
ERM
NHE1
PAK
IRSp53
WAVE2
VCL
ACTN
PIP2
PI4P5K
ROCK
Focal adhesion
F-Actin
mDia
MLCP
MLCK
PI4P5K
LIMK
Arp2/3
CFN
Stabilization of actin
PFN
MLC
PIP2
SSH
Lamellipodia
Actin polymerization stress fiber
Actomyosin assembly contraction
F-Actin
VCL
GSN
MyosinII
Stress fibers
Focal complex assembly
Focal adhesion
04810 8/29/14
(c) Kanehisa Laboratories

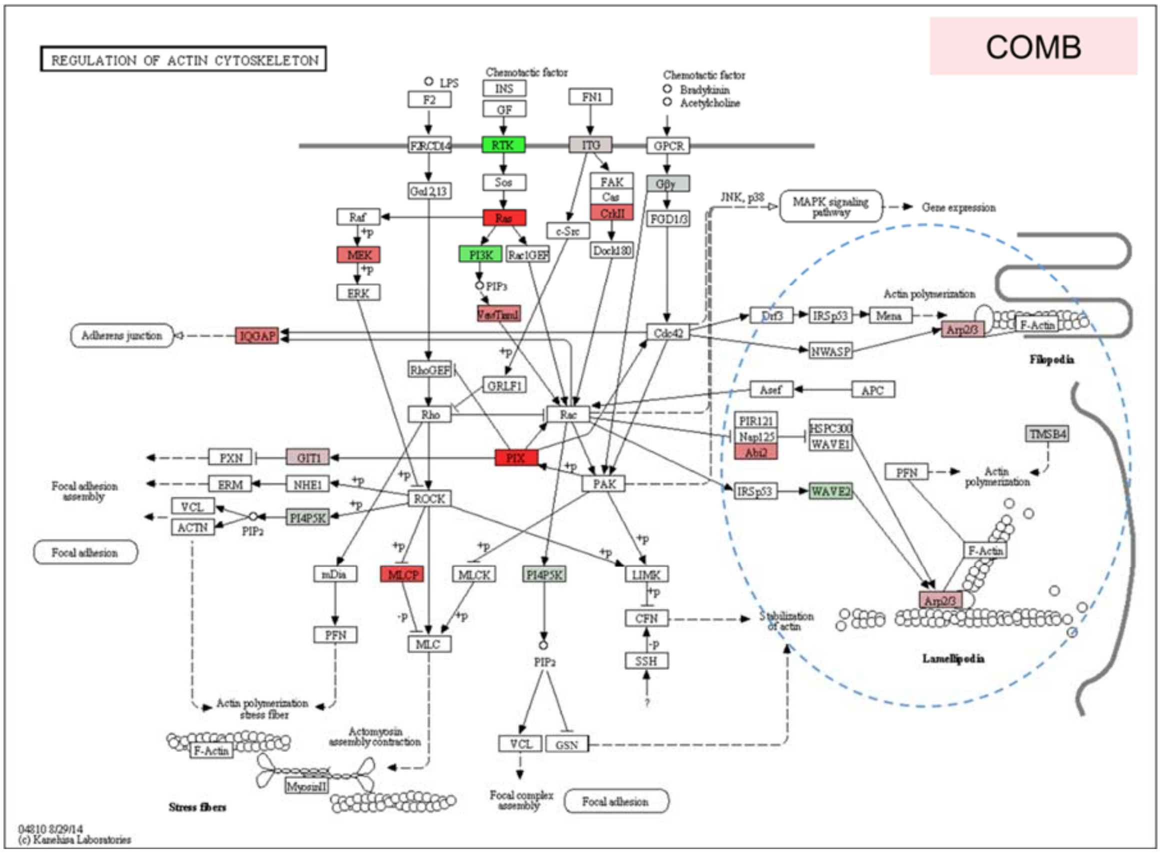
COMB
REGULATION OF ACTIN CYTOSKELETON
LPS
Chemotactic factor
INS
GF
F2
FN1
Chemotactic factor
Bradykinin
Acetylcholine
RTK
ITG
GPCR
Sos
FAK
Cas
CrkII
Ras
Raf
MEK
ERK
PI3K
Rac1GEF
PIP3
c-Src
Dock180
FGD1/3
JNK, p38
MAPK signaling pathway
Gene expression
Adherens junction
IQGAP
Cdc42
Drf3
IRSp53
Mena
Actin polymerization
Arp2/3
F-Actin
Filopodia
NWASP
RhoGEF
GRLF1
Rho
Rac
Asef
APC
PIR121
Nap125
Abi2
HSPC300
WAVE1
TMSB4
PXN
GIT1
PIX
PAK
PFN
Actin polymerization
Focal adhesion assembly
ERM
NHE1
ROCK
IRSp53
WAVE2
VCL
ACTN
PIP2
PI4P5K
Focal adhesion
F-Actin
mDia
MLCP
MLCK
PI4P5K
LIMK
CFN
Stabilization of actin
Arp2/3
PFN
MLC
PIP2
SSH
Lamellipodia
Actin polymerization stress fiber
Actomyosin assembly contraction
F-Actin
VCL
GSN
MyosinII
Stress fibers
Focal complex assembly
Focal adhesion
04810 8/29/14
(c) Kanehisa Laboratories

Figure S-5. Volcano plots illustrating the changes of protein abundance in the control group at each time point. The *x* axis shows the ratio of protein abundance in control group at each time point normalized by time zero transformed by *log 2*, while the y axis shows the *p* values transformed by *log 10* for the comparison. Each dot represents a unique protein group and the dashed lines denote the selected cutoff thresholds (p <0.05 and >1.4-fold change in either directions) that define significantly-altered proteins. The *red* area indicates significant increase and the *green* area indicates significant decrease. The percentage of significantly-changed proteins at each protein was calculated at the bottom.

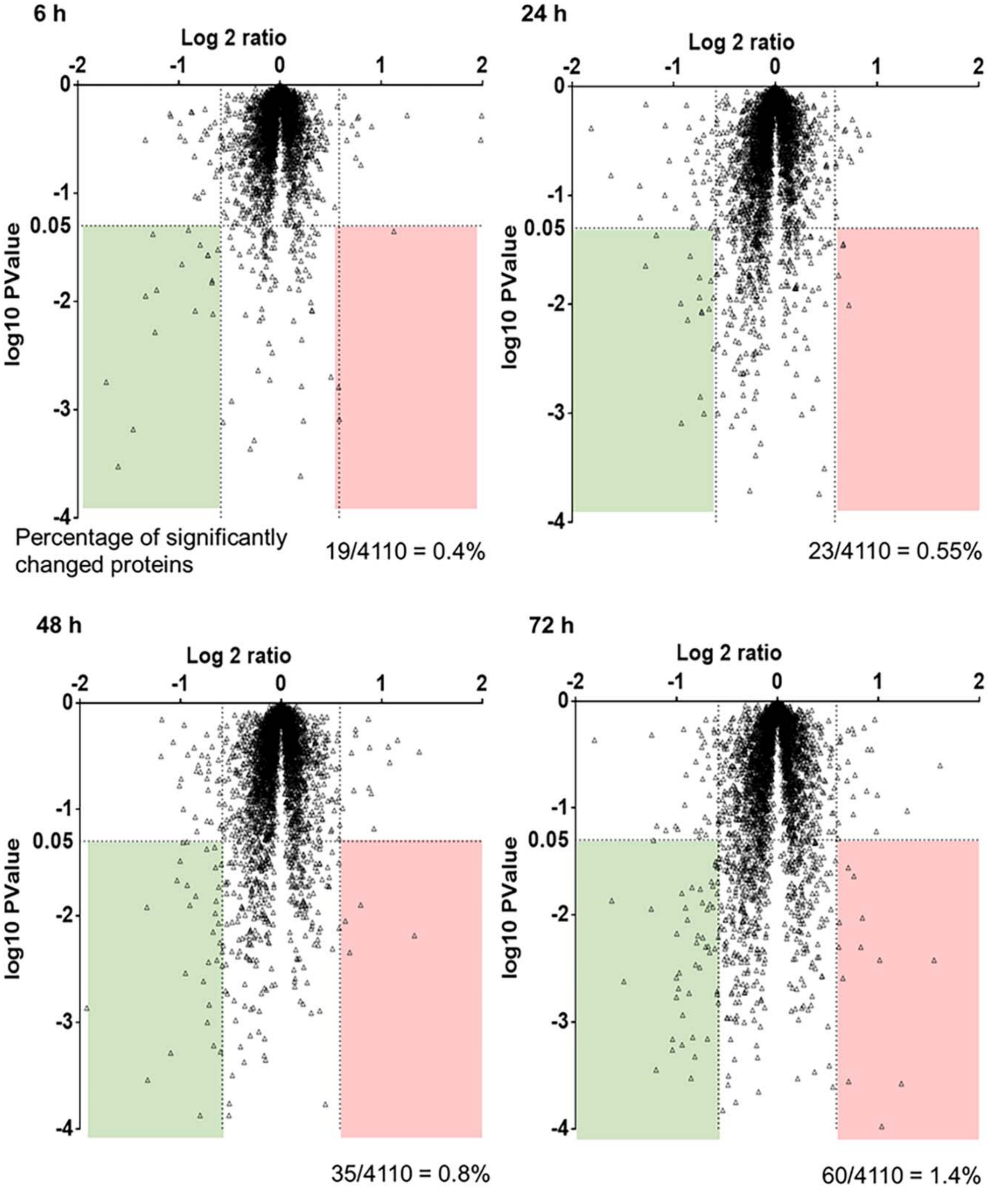
6 h
Log 2 ratio
log10 PValue
0.05
Percentage of significantly changed proteins
19/4110 = 0.4%
24 h
Log 2 ratio
log10 PValue
0.05
23/4110 = 0.55%
48 h
Log 2 ratio
log10 PValue
0.05
35/4110 = 0.8%
72 h
Log 2 ratio
log10 PValue
0.05
60/4110 = 1.4%

Table S-1. List of UniProt ID, gene names, and protein names corresponding to the heat maps (Figure 3A, 3C, 3D, 5A, 5B, 6A, 6B).

Proteins Regulating Cell Cycle Progression		
**Uniprot ID**	**Gene Name**	**Proteins**
P11802	CDK4	Cyclin-dependent kinase 4 (CDK4)
P06400	RB1	Retinoblastoma-associated protein (protein Rb)
P24941	CDK2	Cyclin-dependent kinase 2 (CDK2)
Q14566	MCM6	DNA replication licensing factor MCM6
O43913	ORC5	Origin recognition complex subunit 5
P06493	CDK1	Cyclin-dependent kinase 1 (CDK1)
P14635	CCNB1	G2/mitotic-specific cyclin-B1
O95067	CCNB2	G2/mitotic-specific cyclin-B2
Q99640	PKMYT1	Membrane-associated tyrosine- and threonine-specific cdc2-inhibitory kinase (Myt1 kinase)
P53350	PLK1	Serine/threonine-protein kinase PLK1
P33981	TTK	Dual specificity protein kinase TTK (Phosphotyrosine picked threonine-protein kinase)
Q9Y6D9	MAD1L1	Mitotic spindle assembly checkpoint protein MAD1
Q13257	MAD2L1	Mitotic spindle assembly checkpoint protein MAD2A
O60566	BUB1B	Mitotic checkpoint serine/threonine-protein kinase BUB1 beta
O43683	BUB1	Mitotic checkpoint serine/threonine-protein kinase BUB1
O43684	BUB3	Mitotic checkpoint protein BUB3
P30260	CDC27	Cell division cycle protein 27 homolog
Q13042	CDC16	Cell division cycle protein 16 homolog
Q12834	CDC20	Cell division cycle protein 20 homolog
Q8N3U4	STAG2	Cohesin subunit SA-2 (SCC3 homolog 2)
P04637	TP53	Cellular tumor antigen p53
Q13315	ATM	Serine-protein kinase ATM (Ataxia telangiectasia mutated)
Q92793	CREBBP	CREB-binding protein (p300-CBP coactivator family)
P51946	CCNH	Cyclin-H
P84022	SMAD3	Mothers against decapentaplegic homolog 3
P49841	GSK3B	Glycogen synthase kinase-3 beta (GSK-3 beta)

Proteins Regulating DNA Damage Responses (DDRs)		
**Uniprot ID**	**Gene Name**	**Proteins**
O14974	PPP1R12A	Protein phosphatase 1 regulatory subunit 12A
P60510	PPP4C	Serine/threonine-protein phosphatase 4 catalytic subunit (PP4C)
Q5VTR2	RNF20	E3 ubiquitin-protein ligase BRE1A (BRE1-A)
Q9Y4E8	USP15	Ubiquitin carboxyl-terminal hydrolase 15
O75170	PPP6R2	Serine/threonine-protein phosphatase 6 regulatory subunit 2
O75688	PPM1B/ PP2CB	Protein phosphatase 1B
P53041	PPP5C	Serine/threonine-protein phosphatase 5 (PP5)
Q13315	ATM	Serine-protein kinase ATM (Ataxia telangiectasia mutated)
Q15257	PPP2R4	Serine/threonine-protein phosphatase 2A activator
Q15648	MED1	Mediator of RNA polymerase II transcription subunit 1
Q6NYC8	PPP1R18	Phostensin (Protein phosphatase 1 regulatory subunit 18)
Q8TF42	UBASH3B	Ubiquitin-associated and SH3 domain-containing protein B
Q96A00	PPP1R14A	Protein phosphatase 1 regulatory subunit 14A
Q96C90	PPP1R14B	Protein phosphatase 1 regulatory subunit 14B
Q9BZL4	PPP1R12C	Protein phosphatase 1 regulatory subunit 12C
Q9H2K8	TAOK3	Serine/threonine-protein kinase TAO3 (Thousand and one amino acid protein 3)

DNA Repair Proteins		
**Uniprot ID**	**Gene Name**	**Proteins**
P11388	TOP2A	DNA topoisomerase 2-alpha
P51946	CCNH	Cyclin-H
Q14807	KIF22	Kinesin-like protein KIF22 (Kinesin-like DNA-binding protein)
Q13315	ATM	Serine-protein kinase ATM (Ataxia telangiectasia mutated)
Q9NWV8	BABAM1	BRISC and BRCA1-A complex member 1
P35251	RFC1	Replication factor C subunit 1
P28340	POLD1	DNA polymerase delta catalytic subunit
Q15054	POLD3	DNA polymerase delta subunit 3
Q9NRD1	FBXO6	F-box only protein 6
O15457	MSH4	MutS protein homolog 4 (hMSH4)
Q96EB6	SIRT1	NAD-dependent protein deacetylase sirtuin-1 (hSIRT1)
Q96T76	MMS19	MMS19 nucleotide excision repair protein homolog (hMMS19)
Q9NXR7	BRE	BRCA1-A complex subunit BRE
P04637	TP53	Cellular tumor antigen p53
Q7Z2E3	APTX	Aprataxin
Q8IY18	SMC5	Structural maintenance of chromosomes protein 5 (SMC-5)
P26583	HMGB2	High mobility group protein B2 (HMG-2)
Q7LG56	RRM2B	Ribonucleoside-diphosphate reductase subunit M2 B
P40937	RFC5	Replication factor C subunit 5
O00255	MEN1	Menin
P36639	NUDT1	7,8-dihydro-8-oxoguanine triphosphatase (Nucleoside diphosphate-linked moiety X motif 1) (Nudix motif 1)
P09429	HMGB1	High mobility group protein B1 (HMG-1)
P43246	MSH2	DNA mismatch repair protein Msh2
Q13112	CHAF1B	Chromatin assembly factor 1 subunit B (CAF-1 subunit B)
P29590	PML	Protein PML
P00441	SOD1	Superoxide dismutase 1 (hSod1)
P48730	CSNK1D	Casein kinase I isoform delta (CKI-delta)
P35250	RFC2	Replication factor C subunit 2
Q13426	XRCC4	DNA repair protein XRCC4 (X-ray repair cross-complementing protein 4)
P51948	MNAT1	CDK-activating kinase assembly factor MAT1 (CDK7/cyclin-H assembly factor)
Q9HCS7	XAB2	Pre-mRNA-splicing factor SYF1 (Protein HCNP)
Q15014	MORF4L2	Mortality factor 4-like protein 2
P54725	RAD23A	UV excision repair protein RAD23 homolog A (HR23A)
P04818	TYMS	Thymidylate synthase
Q9UKK3	PARP4	Poly [ADP-ribose] polymerase 4 (PARP-4)
Q9HAW4	CLSPN	Claspin
P18887	XRCC1	DNA repair protein XRCC1

Pro-Apoptotic Proteins		
**Uniprot ID**	**Gene Name**	**Proteins**
P06400	RB1	Retinoblastoma-associated protein (protein Rb)
P04637	TP53	Cellular tumor antigen p53
Q13625	TP53BP2	Apoptosis-stimulating of p53 protein 2 (Bcl2-binding protein)
Q00535	CDK5	Cyclin-dependent-like kinase 5
P22570	FDXR	NADPH:adrenodoxin oxidoreductase, mitochondrial
Q96IZ0	PAWR	PRKC apoptosis WT1 regulator protein
Q16611	BAK1	Bcl-2 homologous antagonist/killer (Apoptosis regulator BAK)
P61769	B2M	Beta-2-microglobulin
P05067	APP	Amyloid beta A4 protein
P29590	PML	Protein PML (Promyelocytic leukemia protein)
Q99643	SDHC	Succinate dehydrogenase cytochrome b560 subunit, mitochondrial
Q13257	MAD2L1	Mitotic spindle assembly checkpoint protein MAD2A
Q96FV9	THOC1	THO complex subunit 1 (Tho1)
P84022	SMAD3	Mothers against decapentaplegic homolog 3 (MAD homolog 3)
O60831	PRAF2	PRA1 family protein 2
P10909	CLU	Clusterin
P06493	CDK1	Cyclin-dependent kinase 1 (CDK1)
Q9ULZ3	PYCARD	Apoptosis-associated speck-like protein containing a CARD
P25445	FAS	Tumor necrosis factor receptor superfamily member 6 (Apoptosis-mediating surface antigen FAS, CD95)
P11388	TOP2A	DNA topoisomerase 2-alpha
P23921	RRM1	Ribonucleoside-diphosphate reductase large subunit
Q9Y6K5	OAS3	2'-5'-oligoadenylate synthase 3
Q07812	BAX	Apoptosis regulator BAX
P49815	TSC2	Tuberin

Anti-Apoptotic Proteins		
**Uniprot ID**	**Gene Name**	**Proteins**
P01112	HRAS	GTPase HRas (H-Ras-1)
P07203	GPX1	Glutathione peroxidase 1 (GPx-1)
P14635	CCNB1	G2/mitotic-specific cyclin-B1
P78536	ADAM17	Disintegrin and metalloproteinase domain-containing protein 17 (ADAM 17)
Q02750	MAP2K1	Dual specificity mitogen-activated protein kinase kinase 1 (MAP kinase kinase 1) (MAPKK 1) (MKK1)
Q9Y6A5	TACC3	Transforming acidic coiled-coil-containing protein 3
P53350	PLK1	Serine/threonine-protein kinase PLK1
P53611	RABGGTB	Geranylgeranyl transferase type-2 subunit beta
P06454	PTMA	Prothymosin alpha
P01111	NRAS	GTPase NRas (Transforming protein N-Ras)
O00762	UBE2C	Ubiquitin-conjugating enzyme E2 C
Q8WXI7	MUC16	Mucin-16 (MUC-16)
O15392	BIRC5	Baculoviral IAP repeat-containing protein 5 (Survivin)
Q9P2P6	STARD9	StAR-related lipid transfer protein 9 (StARD9)
Q96EB6	SIRT1	NAD-dependent protein deacetylase sirtuin-1(hSIRT1)
O94763	URI1	Unconventional prefoldin RPB5 interactor 1 (Protein NNX3)
P09211	GSTP1	Glutathione S-transferase P
O15439	ABCC4	Multidrug resistance-associated protein 4 (ATP-binding cassette sub-family C member 4)
O75475	PSIP1	PC4 and SFRS1-interacting protein
O75934	BCAS2	Pre-mRNA-splicing factor SPF27 (Breast carcinoma-amplified sequence 2)
P16144	ITGB4	Integrin beta-4
P19174	PLCG1	1-phosphatidylinositol 4,5-bisphosphate phosphodiesterase gamma-1 (PLC-gamma-1)
P37268	FDFT1	Squalene synthase (Farnesyl-diphosphate farnesyltransferase)
P41743	PRKCI	Protein kinase C iota type
P42345	MTOR	Serine/threonine-protein kinase mTOR
P49327	FASN	Fatty acid synthase
P49711	CTCF	Transcriptional repressor CTCF
Q13315	ATM	Serine-protein kinase ATM (Ataxia telangiectasia mutated)
Q13740	ALCAM	CD166 antigen (Activated leukocyte cell adhesion molecule)
Q14318	FKBP8	Peptidyl-prolyl cis-trans isomerase FKBP8 (PPIase FKBP8)
Q15058	KIF14	Kinesin-like protein KIF14
Q3YEC7	RABL6	Rab-like protein 6 (GTP-binding protein Parf)
Q8TDX7	NEK7	Serine/threonine-protein kinase Nek7
Q92696	RABGGTA	Geranylgeranyl transferase type-2 subunit alpha
Q96Q42	ALS2	Alsin (Amyotrophic lateral sclerosis 2 chromosomal region candidate gene 6 protein)
Q96RE7	NACC1	Nucleus accumbens-associated protein 1 (NAC-1)
Q99496	RNF2	E3 ubiquitin-protein ligase RING2
Q9H2K8	TAOK3	Serine/threonine-protein kinase TAO3 (Thousand and one amino acid protein 3)
Q9NR09	BIRC6	Baculoviral IAP repeat-containing protein 6 (BRUCE)
Q9UHD2	TBK1	Serine/threonine-protein kinase TBK1

Pro-Migration Proteins		
**Uniprot ID**	**Gene Name**	**Proteins**
P52292	KPNA2	Importin subunit alpha-1 (Karyopherin subunit alpha-2)
P78536	ADAM17	Disintegrin and metalloproteinase domain-containing protein 17 (ADAM 17)
P17813	ENG	Endoglin (CD antigen CD105)
P46108	CRK	Adapter molecule crk (Proto-oncogene c-Crk)
P51858	HDGF	Hepatoma-derived growth factor (HDGF)
Q13740	ALCAM	CD166 antigen (Activated leukocyte cell adhesion molecule)
O94842	TOX4	TOX high mobility group box family member 4 (Epidermal Langerhans cell protein LCP1)
Q9UBR2	CTSZ	Cathepsin Z
Q13136	PPFIA1	Liprin-alpha-1 (LAR-interacting protein 1) (LIP-1)
P19174	PLCG1	1-phosphatidylinositol 4,5-bisphosphate phosphodiesterase gamma-1 (PLC-gamma-1)
P15144	ANPEP	Aminopeptidase N
P06756	ITGAV	Integrin alpha-V
P18084	ITGB5	Integrin beta-5
O60488	ACSL4	Long-chain-fatty-acid--CoA ligase 4 (LACS 4)
Q00535	CDK5	Cyclin-dependent-like kinase 5
O14745	SLC9A3R1	Na(+)/H(+) exchange regulatory cofactor NHE-RF1 (NHERF-1)
Q93008	USP9X	Probable ubiquitin carboxyl-terminal hydrolase FAF-X
P55011	SLC12A2	Solute carrier family 12 member 2
P41743	PRKCI	Protein kinase C iota type
Q9Y2X7	GIT1	ARF GTPase-activating protein GIT1
P34741	SDC2	Syndecan-2
Q96EB6	SIRT1	NAD-dependent protein deacetylase sirtuin-1 (hSIRT1)
Q16787	LAMA3	Laminin subunit alpha-3
P30530	AXL	Tyrosine-protein kinase receptor UFO
P98170	XIAP	E3 ubiquitin-protein ligase XIAP (X-linked IAP)
P11166	SLC2A1	Solute carrier family 2, facilitated glucose transporter member 1
O00622	CYR61	Protein CYR61 (Insulin-like growth factor-binding protein 10)
P49841	GSK3B	Glycogen synthase kinase-3 beta (GSK-3 beta)
P41134	ID1	DNA-binding protein inhibitor ID-1
P05121	SERPINE1	Plasminogen activator inhibitor 1 (PAI-1)

Pro-Invasion Proteins		
Uniprot ID	Gene Name	Proteins
P11166	SLC2A1	Solute carrier family 2, facilitated glucose transporter member 1
P54760	EPHB4	Ephrin type-B receptor 4 (Tyrosine-protein kinase TYRO11)
P29317	EPHA2	Ephrin type-A receptor 2 (Tyrosine-protein kinase receptor ECK)
P41134	ID1	DNA-binding protein inhibitor ID-1 (Inhibitor of DNA binding 1)
P00533	EGFR	Epidermal growth factor receptor (Receptor tyrosine-protein kinase erbB-1)
P29323	EPHB2	Ephrin type-B receptor 2 (Tyrosine-protein kinase TYRO5)
P98170	XIAP	E3 ubiquitin-protein ligase XIAP (X-linked IAP)
P06703	S100A6	Protein S100-A6 (Calcyclin)
P42684	ABL2	Abelson tyrosine-protein kinase 2 (Tyrosine-protein kinase ARG)
P34741	SDC2	Syndecan-2 (SYND2) (Fibroglycan)
P78325	ADAM8	Disintegrin and metalloproteinase domain-containing protein 8 (ADAM 8)
O14786	NRP1	Neuropilin-1
Q13740	ALCAM	CD166 antigen (Activated leukocyte cell adhesion molecule)
P16144	ITGB4	Integrin beta-4
Q9Y5S2	CDC42BPB	Serine/threonine-protein kinase MRCK beta
P55011	SLC12A2	Solute carrier family 12 member 2
P51946	CCNH	Cyclin-H
Q86Y07	VRK2	Serine/threonine-protein kinase VRK2
P19174	PLCG1	1-phosphatidylinositol 4,5-bisphosphate phosphodiesterase gamma-1 (PLC-gamma-1)
Q9Y6W5	WASF2	Wiskott-Aldrich syndrome protein family member 2 (Protein WAVE-2)
Q13443	ADAM9	Disintegrin and metalloproteinase domain-containing protein 9 (ADAM 9)
O60488	ACSL4	Long-chain-fatty-acid--CoA ligase 4 (LACS 4)
P42345	MTOR	Serine/threonine-protein kinase mTOR
P46937	YAP1	Transcriptional coactivator YAP1
P51858	HDGF	Hepatoma-derived growth factor (HDGF)
O94842	TOX4	TOX high mobility group box family member 4
Q9UBR2	CTSZ	Cathepsin Z
P48960	CD97	CD97 antigen
P09429	HMGB1	High mobility group protein B1 (HMG-1)
Q8IYB3	SRRM1	Serine/arginine repetitive matrix protein 1
Q00535	CDK5	Cyclin-dependent-like kinase 5
P41743	PRKCI	Protein kinase C iota type

Table S-2. Representative raw dataset for Figure 3-6.

UniProt ID	Protein Name	LOG2(fold change normalized by time zero)				
		Gem_0 h	Gem_6 h	Gem_24 h	Gem_48 h	Gem_72 h
P11802	CDK4	0	0.1628	0.1158	-0.4404	-0.5928
P06400	RB1	0	0.1078	0.2678	0.4964	0.4929
P24941	CDK2	0	0.0445	0.1152	0.2287	0.2013
Q14566	MCM6	0	0.0292	0.0783	-0.4873	-0.6183
O43913	ORC5	0	-0.0491	-0.0244	-0.8795	-0.5864
P06493	CDK1	0	0.0557	0.1922	0.7819	0.7150
P14635	CCNB1	0	-0.0702	0.2832	0.8391	-0.0183
O95067	CCNB2	0	-0.3686	0.5803	1.7056	2.6396
Q99640	PKMYT1	0	0.2539	0.2939	0.6950	0.3167
P53350	PLK1	0	-0.0936	0.0686	0.3391	-0.0664
P33981	TTK	0	0.0559	-0.0713	-0.3567	-0.4216
Q9Y6D9	MAD1L1	0	-0.0444	-0.2030	-0.8098	-0.9672
Q13257	MAD2L1	0	0.0312	0.2098	-0.5573	-0.5421
O60566	BUB1B	0	0.5298	1.3722	2.4057	1.6429
O43683	BUB1	0	-0.2673	0.0816	0.4957	0.1208
O43684	BUB3	0	0.1379	0.0501	0.6006	0.5166
P30260	CDC27	0	-0.1995	-0.1857	0.8349	0.7382
Q13042	CDC16	0	-0.1342	-0.1055	0.5328	0.7322
Q12834	CDC20	0	-0.4214	-0.2007	-0.1215	-0.0484
Q8N3U4	STAG2	0	0.1610	0.3574	-0.6111	-0.4463
P04637	TP53	0	0.0558	0.1504	0.5448	0.2310
Q13315	ATM	0	0.0418	-0.1057	0.9472	1.0234
Q92793	CREBBP	0	0.0105	-0.1697	0.6865	0.9515
P51946	CCNH	0	0.1481	0.0636	0.6109	0.6404
P84022	SMAD3	0	0.4339	0.4791	-1.0539	-1.2705
P49841	GSK3B	0	0.0646	0.0270	-0.4265	-0.0111

UniProt ID	Protein Name	LOG2(fold change normalized by time zero)				
		Bnt_0 h	Bnt_6 h	Bnt_24 h	Bnt_48 h	Bnt_72 h
P11802	CDK4	0	0.0168	-0.0809	-0.0973	-0.0700
P06400	RB1	0	-0.0049	0.2202	0.1902	0.3289
P24941	CDK2	0	0.0173	0.0653	0.1831	0.1247
Q14566	MCM6	0	-0.0139	-0.0891	0.0132	0.0072
O43913	ORC5	0	-0.0191	-0.2795	-0.1385	-0.4394
P06493	CDK1	0	0.0517	0.1509	0.2549	0.1637
P14635	CCNB1	0	0.1152	-0.2014	-0.3193	-0.5021
O95067	CCNB2	0	0.0373	0.3955	0.0699	-1.1353
Q99640	PKMYT1	0	0.6419	0.7422	0.1683	0.0584
P53350	PLK1	0	-0.0271	-0.0928	-0.3198	-0.5566
P33981	TTK	0	0.0377	0.2687	-0.3344	-0.8478
Q9Y6D9	MAD1L1	0	0.1223	-0.0471	0.6377	0.3609
Q13257	MAD2L1	0	-0.1620	0.1372	0.1875	0.0136
O60566	BUB1B	0	-0.7131	-0.7686	-1.1420	-0.9213
O43683	BUB1	0	-0.5383	-0.2617	-1.0983	-1.1386
O43684	BUB3	0	-0.0065	0.0455	-0.0898	-0.1134
P30260	CDC27	0	-0.1449	-0.2904	-0.3083	-0.3871
Q13042	CDC16	0	0.0017	0.0832	0.0132	-0.2482
Q12834	CDC20	0	0.0353	-0.2753	-0.6223	-0.8665
Q8N3U4	STAG2	0	-0.4880	-0.1972	-0.6223	-0.8687
P04637	TP53	0	0.1143	-0.0262	0.2535	0.1886
Q13315	ATM	0	0.1224	-0.7688	-0.0057	-0.1232
Q92793	CREBBP	0	0.0648	-0.0426	-0.0750	-0.3712
P51946	CCNH	0	0.2267	0.1099	-0.1443	0.0887
P84022	SMAD3	0	-0.0344	-0.0307	0.0486	0.2105
P49841	GSK3B	0	-0.2085	-0.4069	-0.4789	-0.3094

UniProt ID	Protein Name	LOG2(fold change normalized by time zero)				
		Comb_0 h	Comb_6 h	Comb_24 h	Comb_48 h	Comb_72 h
P11802	CDK4	0	-0.2136	0.6180	-0.3663	0.6645
P06400	RB1	0	0.0450	0.3059	0.2095	0.2375
P24941	CDK2	0	0.1255	0.1905	0.3950	0.5772
Q14566	MCM6	0	0.0305	0.0613	-0.0657	0.0471
O43913	ORC5	0	-0.2492	-0.7634	-0.4680	-1.0560
P06493	CDK1	0	0.0833	0.3369	0.5603	0.6717
P14635	CCNB1	0	-0.0381	0.5716	0.8596	0.5657
O95067	CCNB2	0	-1.2474	-0.2829	-0.4182	-1.5183
Q99640	PKMYT1	0	0.1178	0.4038	0.7469	0.3250
P53350	PLK1	0	0.0980	0.2662	0.3638	0.2274
P33981	TTK	0	0.0925	0.5440	0.3840	0.9445
Q9Y6D9	MAD1L1	0	0.1551	0.2418	0.4566	0.3399
Q13257	MAD2L1	0	0.0669	0.3277	0.5035	-0.0102
O60566	BUB1B	0	0.0545	0.4861	0.9145	0.7894
O43683	BUB1	0	-0.3734	0.0994	-0.0121	-0.0662
O43684	BUB3	0	-0.0139	-0.1233	-0.1337	-0.1354
P30260	CDC27	0	-0.1548	-0.1130	0.2443	0.0888
Q13042	CDC16	0	0.9712	-0.8054	-0.7637	-0.0772
Q12834	CDC20	0	-0.0630	0.1469	0.1626	0.0815
Q8N3U4	STAG2	0	-0.3212	-0.2678	-0.2961	-0.5960
P04637	TP53	0	-0.0563	0.0312	0.1964	0.0838
Q13315	ATM	0	0.1297	0.1281	0.2032	0.3504
Q92793	CREBBP	0	-0.0315	-0.0735	-0.2048	-0.6109
P51946	CCNH	0	-0.3154	-0.0190	-0.2106	-0.4759
P84022	SMAD3	0	0.0037	-0.0947	0.0612	0.2146
P49841	GSK3B	0	-0.1141	0.0726	-0.5609	-0.8549
